# Scientists’ warning to humanity: microorganisms and climate change

**DOI:** 10.1038/s41579-019-0222-5

**Published:** 2019-06-18

**Authors:** Ricardo Cavicchioli, William J. Ripple, Kenneth N. Timmis, Farooq Azam, Lars R. Bakken, Matthew Baylis, Michael J. Behrenfeld, Antje Boetius, Philip W. Boyd, Aimée T. Classen, Thomas W. Crowther, Roberto Danovaro, Christine M. Foreman, Jef Huisman, David A. Hutchins, Janet K. Jansson, David M. Karl, Britt Koskella, David B. Mark Welch, Jennifer B. H. Martiny, Mary Ann Moran, Victoria J. Orphan, David S. Reay, Justin V. Remais, Virginia I. Rich, Brajesh K. Singh, Lisa Y. Stein, Frank J. Stewart, Matthew B. Sullivan, Madeleine J. H. van Oppen, Scott C. Weaver, Eric A. Webb, Nicole S. Webster

**Affiliations:** 1 School of Biotechnology and Biomolecular Sciences, The University of New South Wales, Sydney, NSW Australia; 2 Department of Forest Ecosystems and Society, Oregon State University, Corvallis, OR USA; 3 Institute of Microbiology, Technical University Braunschweig, Braunschweig, Germany; 4 Scripps Institution of Oceanography, University of California San Diego, La Jolla, CA USA; 5 Faculty of Chemistry, Biotechnology and Food Science, Norwegian University of Life Sciences, Ås, Norway; 6 Institute of Infection and Global Health, University of Liverpool, Liverpool, UK; 7 Department of Botany and Plant Pathology, Oregon State University, Corvallis, OR USA; 8 Alfred Wegener Institute, Helmholtz Center for Marine and Polar Research, Bremerhaven, Germany; 9 Max Planck Institute for Marine Microbiology, Bremen, Germany; 10 Institute for Marine and Antarctic Studies, University of Tasmania, Hobart, TAS Australia; 11 Rubenstein School of Environment and Natural Resources, and The Gund Institute for Environment, University of Vermont, Burlington, VT USA; 12 Institute of Integrative Biology, ETH Zurich, Zurich, Switzerland; 13 Department of Life and Environmental Sciences, Polytechnic University of Marche, Ancona, Italy; 14 Stazione Zoologica Anton Dohrn, Naples, Italy; 15 Center for Biofilm Engineering, and Chemical and Biological Engineering Department, Montana State University, Bozeman, MT USA; 16 Department of Freshwater and Marine Ecology, Institute for Biodiversity and Ecosystem Dynamics, University of Amsterdam, Amsterdam, Netherlands; 17 Department of Biological Sciences, Marine and Environmental Biology Section, University of Southern California, Los Angeles, CA USA; 18 Biological Sciences Division, Earth and Biological Sciences Directorate, Pacific Northwest National Laboratory, Richland, WA USA; 19 Daniel K. Inouye Center for Microbial Oceanography: Research and Education, School of Ocean and Earth Science & Technology, University of Hawaii at Manoa, Honolulu, HI USA; 20 Department of Integrative Biology, University of California, Berkeley, Berkeley, CA USA; 21 Marine Biological Laboratory, Woods Hole, MA USA; 22 Department of Ecology and Evolutionary Biology, University of California, Irvine, Irvine, CA USA; 23 Department of Marine Sciences, University of Georgia, Athens, GA USA; 24 Division of Geological and Planetary Sciences, California Institute of Technology, Pasadena, CA USA; 25 School of Geosciences, University of Edinburgh, Edinburgh, UK; 26 Division of Environmental Health Sciences, School of Public Health, University of California, Berkeley, Berkeley, CA USA; 27 Microbiology Department, and the Byrd Polar and Climate Research Center, The Ohio State University, Columbus, OH USA; 28 Hawkesbury Institute for the Environment, and Global Centre for Land-Based Innovation, Western Sydney University, Penrith, NSW Australia; 29 Department of Biological Sciences, University of Alberta, Edmonton, AB Canada; 30 School of Biological Sciences, Georgia Institute of Technology, Atlanta, GA USA; 31 Department of Microbiology, and Department of Civil, Environmental and Geodetic Engineering, and the Byrd Polar and Climate Research Center, The Ohio State University, Columbus, OH USA; 32 School of BioSciences, The University of Melbourne, Parkville, VIC Australia; 33 Australian Institute of Marine Science, Townsville, QLD Australia; 34 Department of Microbiology and Immunology, and Institute for Human Infections and Immunity, University of Texas Medical Branch, Galveston, TX USA; 35 Australian Centre for Ecogenomics, University of Queensland, Brisbane, QLD Australia

**Keywords:** Environmental microbiology, Microbial ecology, Biogeochemistry, Climate-change impacts, Climate-change adaptation, Climate-change mitigation, Ecosystem services, Infectious diseases

## Abstract

In the Anthropocene, in which we now live, climate change is impacting most life on Earth. Microorganisms support the existence of all higher trophic life forms. To understand how humans and other life forms on Earth (including those we are yet to discover) can withstand anthropogenic climate change, it is vital to incorporate knowledge of the microbial ‘unseen majority’. We must learn not just how microorganisms affect climate change (including production and consumption of greenhouse gases) but also how they will be affected by climate change and other human activities. This Consensus Statement documents the central role and global importance of microorganisms in climate change biology. It also puts humanity on notice that the impact of climate change will depend heavily on responses of microorganisms, which are essential for achieving an environmentally sustainable future.

## Abstract (web summary)

The microbial majority with which we share Earth often goes unnoticed despite underlying major biogeochemical cycles and food webs, thereby taking a key role in climate change. This Consensus Statement highlights the importance of climate change microbiology and issues a call to action for all microbiologists.

## Introduction

Human activities and their effects on the climate and environment cause unprecedented animal and plant extinctions, cause loss in biodiversity^1–4^ and endanger animal and plant life on Earth^5^. Losses of species, communities and habitats are comparatively well researched, documented and publicized^6^. By contrast, microorganisms are generally not discussed in the context of climate change (particularly the effect of climate change on microorganisms). While invisible to the naked eye and thus somewhat intangible^7^, the abundance (~10^30^ total bacteria and archaea)^8^ and diversity of microorganisms underlie their role in maintaining a healthy global ecosystem: simply put, the microbial world constitutes the life support system of the biosphere. Although human effects on microorganisms are less obvious and certainly less characterized, a major concern is that changes in microbial biodiversity and activities will affect the resilience of all other organisms and hence their ability to respond to climate change^9^.

Microorganisms have key roles in carbon and nutrient cycling, animal (including human) and plant health, agriculture and the global food web. Microorganisms live in all environments on Earth that are occupied by macroscopic organisms, and they are the sole life forms in other environments, such as the deep subsurface and ‘extreme’ environments. Microorganisms date back to the origin of life on Earth at least 3.8 billion years ago, and they will likely exist well beyond any future extinction events.

Although microorganisms are crucial in regulating climate change, they are rarely the focus of climate change studies and are not considered in policy development. Their immense diversity and varied responses to environmental change make determining their role in the ecosystem challenging. In this Consensus Statement, we illustrate the links between microorganisms, macroscopic organisms and climate change, and put humanity on notice that the microscopic majority can no longer be the unseen elephant in the room. Unless we appreciate the importance of microbial processes, we fundamentally limit our understanding of Earth’s biosphere and response to climate change and thus jeopardize efforts to create an environmentally sustainable future^6^ (Box 1).

Box 1 Scientists’ warning The Alliance of World Scientists and the Scientists’ Warning movement was established to alert humanity to the impacts of human activities on global climate and the environment. In 1992, 1,700 scientists signed the first warning, raising awareness that human impact puts the future of the living world at serious risk^267^. In 2017, 25 years later, the second warning was issued in a publication signed by more than 15,000 scientists^5^. The movement has continued to grow, with more than 21,000 scientists endorsing the warning. At the heart of the warning is a call for governments and institutions to shift policy away from economic growth and towards a conservation economy that will stop environmental destruction and enable human activities to achieve a sustainable future^268^. Linked to the second warning is a series of articles that will focus on specific topics, the first of which describes the importance of conserving wetlands^269^. A film, The Second Warning, also aims to document scientists’ advocacy for humanity to replace ‘business as usual’ and take action to achieve the survival of all species by averting the continuing environmental and climate change crisis.

Complementing the goals of the Alliance of World Scientists are the United Nations Sustainable Development Goals, which were formulated to realize dignity, peace and prosperity for people and the planet, now and into the future^6^. The goals are framed around environmental, economic and social needs, and address sustainability through the elimination of poverty, development of safe cities and educated populations, implementation of renewables (energy generation and consumption) and urgent action on climate change involving equitable use of aquatic and terrestrial systems to achieve a healthy, less polluted biosphere. The goals recognize that responsible management of finite natural resources is required for the development of resilient, sustainable societies.

Our Consensus Statement represents a warning to humanity from the perspective of microbiology. As a microbiologists’ warning, the intent is to raise awareness of the microbial world and make a call to action for microbiologists to become increasingly engaged in and for microbial research to become increasingly integrated into the frameworks for addressing climate change and accomplishing the United Nations Sustainable Development Goals (Box 2). It builds on previous science and policy efforts to call attention to the role of microorganisms in climate change^7,126,270–272^ and their broad relevance to society^7^. Microbiologists are able to endorse the microbiologists’ warning by becoming a signatory.

## Scope of the Consensus Statement

In this Consensus Statement, we address the effects of microorganisms on climate change, including microbial climate-active processes and their drivers. We also address the effects of climate change on microorganisms, focusing on the influences of climate change on microbial community composition and function, physiological responses and evolutionary adaptation. Although we focus on microorganism–climate connections, human activities with a less direct but possibly synergistic effect, such as via local pollution or eutrophication, are also addressed.

For the purpose of this Consensus Statement, we define ‘microorganism’ as any microscopic organism or virus not visible to the naked eye (smaller than 50 μm) that can exist in a unicellular, multicellular (for example, differentiating species), aggregate (for example, biofilm) or viral form. In addition to microscopic bacteria, archaea, eukaryotes and viruses, we discuss certain macroscopic unicellular eukaryotes (for example, larger marine phytoplankton) and wood-decomposing fungi. Our intent is not to exhaustively cover all environments nor all anthropogenic influences but to provide examples from major global biomes (marine and terrestrial) that highlight the effects of climate change on microbial processes and the consequences. We also highlight agriculture and infectious diseases and the role of microorganisms in climate change mitigation. Our Consensus Statement alerts microbiologists and non-microbiologists to address the roles of microorganisms in accelerating or mitigating the impacts of anthropogenic climate change (Box 1).

## Marine biome

**Fig. 1:**
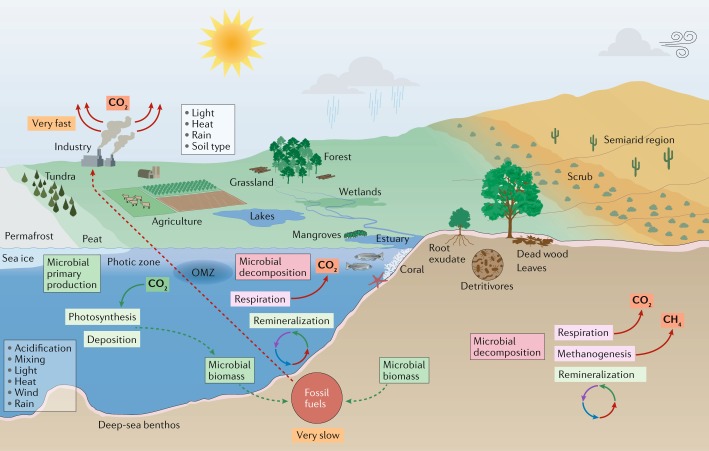
Microorganisms and climate change in marine and terrestrial biomes. In marine environments, microbial primary production contributes substantially to CO_2_ sequestration. Marine microorganisms also recycle nutrients for use in the marine food web and in the process release CO_2_ to the atmosphere. In a broad range of terrestrial environments, microorganisms are the key decomposers of organic matter and release nutrients in the soil for plant growth as well as CO_2_ and CH_4_ into the atmosphere. Microbial biomass and other organic matter (remnants of plants and animals) are converted to fossil fuels over millions of years. By contrast, burning of fossil fuels liberates greenhouse gases in a small fraction of that time. As a result, the carbon cycle is extremely out of balance, and atmospheric CO_2_ levels will continue to rise as long as fossil fuels continue to be burnt. The many effects of human activities, including agriculture, industry, transport, population growth and human consumption, combined with local environmental factors, including soil type and light, greatly influence the complex network of microbial interactions that occur with other microorganisms, plants and animals. These interactions dictate how microorganisms respond to and affect climate change (for example, through greenhouse gas emissions) and how climate change (for example, higher CO_2_ levels, warming, and precipitation changes) in turn affect microbial responses. OMZ, oxygen minimum zone.

Marine biomes cover ~70% of Earth’s surface and range from coastal estuaries, mangroves and coral reefs to the open oceans (Fig. 1). Phototrophic microorganisms use the sun’s energy in the top 200 m of the water column, whereas marine life in deeper zones uses organic and inorganic chemicals for energy^10^. In addition to sunlight, the availability of other energy forms and water temperature (ranging from approximately −2 °C in ice-covered seas to more than 100 °C in hydrothermal vents) influence the composition of marine communities^11^. Rising temperatures not only affect biological processes but also reduce water density and thereby stratification and circulation, which affect organismal dispersal and nutrient transport. Precipitation, salinity and winds also affect stratification, mixing and circulation. Nutrient inputs from air, river and estuarine flows also affect microbial community composition and function, and climate change affects all these physical factors.

The overall relevance of microorganisms to ocean ecosystems can be appreciated from their number and biomass in the water column and subsurface: the total number of cells is more than 10^29^ (refs^8,12–16^) and the Census of Marine Life estimates that 90% of marine biomass is microbial. Beyond their sheer numbers, marine microorganisms fulfil key ecosystem functions. By fixing carbon and nitrogen, and remineralizing organic matter, marine microorganisms form the basis of ocean food webs and thus global carbon and nutrient cycles^13^. The sinking, deposition and burial of fixed carbon in particulate organic matter to marine sediments is a key, long-term mechanism for sequestering CO_2_ from the atmosphere. Therefore, the balance between regeneration of CO_2_ and nutrients via remineralization versus burial in the seabed determines the effect on climate change.

In addition to getting warmer (from increased atmospheric CO_2_ concentrations enhancing the greenhouse effect), oceans have acidified by ~0.1 pH units since preindustrial times, with further reductions of 0.3–0.4 units predicted by the end of the century^17–19^. Given the unprecedented rate of pH change^19–21^, there is a need to rapidly learn how marine life will respond^22^. The impact of elevated greenhouse gas concentrations on ocean temperature, acidification, stratification, mixing, thermohaline circulation, nutrient supply, irradiation and extreme weather events affects the marine microbiota in ways that have substantial environmental consequences, including major shifts in productivity, marine food webs, carbon export and burial in the seabed^19,23–29^.

### Microorganisms affect climate change

Marine phytoplankton perform half of the global photosynthetic CO_2_ fixation (net global primary production of ~50 Pg C per year) and half of the oxygen production despite amounting to only ~1% of global plant biomass^30^. In comparison with terrestrial plants, marine phytoplankton are distributed over a larger surface area, are exposed to less seasonal variation and have markedly faster turnover rates than trees (days versus decades)^30^. Therefore, phytoplankton respond rapidly on a global scale to climate variations. These characteristics are important when one is evaluating the contributions of phytoplankton to carbon fixation and forecasting how this production may change in response to perturbations. Predicting the effects of climate change on primary productivity is complicated by phytoplankton bloom cycles that are affected by both bottom-up control (for example, availability of essential nutrients and vertical mixing) and top-down control (for example, grazing and viruses)^27,30–34^. Increases in solar radiation, temperature and freshwater inputs to surface waters strengthen ocean stratification and consequently reduce transport of nutrients from deep water to surface waters, which reduces primary productivity^30,34,35^. Conversely, rising CO_2_ levels can increase phytoplankton primary production, but only when nutrients are not limiting^36–38^.

Some studies indicate that overall global oceanic phytoplankton density has decreased in the past century^39^, but these conclusions have been questioned because of the limited availability of long-term phytoplankton data, methodological differences in data generation and the large annual and decadal variability in phytoplankton production^40–43^. Moreover, other studies suggest a global increase in oceanic phytoplankton production^44^ and changes in specific regions or specific phytoplankton groups^45,46^. The global sea ice (Sea Ice Index) is declining, leading to higher light penetration and potentially more primary production^47^; however, there are conflicting predictions for the effects of variable mixing patterns and changes in nutrient supply and for productivity trends in polar zones^34^. This highlights the need to collect long-term data on phytoplankton production and microbial community composition. Long-term data are needed to reliably predict how microbial functions and feedback mechanisms will respond to climate change, yet only very few such datasets exist (for example, the Hawaii Ocean Time-series and the Bermuda Atlantic Time-series Study)^48–50^. In this context, the Global Ocean Sampling Expedition^51^, transects of the Southern Ocean^52,53^, and the Tara Oceans Consortium^11,54–59^ provide metagenome data that are a valuable baseline of marine microorganisms.

Diatoms perform 25–45% of total primary production in the oceans^60–62^, owing to their prevalence in open-ocean regions when total phytoplankton biomass is maximal^63^. Diatoms have relatively high sinking speeds compared with other phytoplankton groups, and they account for ~40% of particulate carbon export to depth^62,64^. Physically driven seasonal enrichments in surface nutrients favour diatom blooms. Anthropogenic climate change will directly affect these seasonal cycles, changing the timing of blooms and diminishing their biomass, which will reduce primary production and CO_2_ uptake^65^. Remote sensing data suggest a global decline of diatoms between 1998 and 2012, particularly in the North Pacific, which is associated with shallowing of the surface mixed layer and lower nutrient concentrations^46^.

In addition to the contribution of marine phytoplankton to CO_2_ sequestration^30,66–68^, chemolithoautotrophic archaea and bacteria fix CO_2_ under dark conditions in deep ocean waters^69^ and at the surface during polar winter^70^. Marine bacteria and archaea also contribute substantially to surface ocean respiration and cycling of many elements^18^. Seafloor methanogens and methanotrophs are important producers and consumers of CH_4_, but their influence on the atmospheric flux of this greenhouse gas is uncertain^71^. Marine viruses, bacteriovorous bacteria and eukaryotic grazers are also important components of microbial food webs; for example, marine viruses influence how effectively carbon is sequestered and deposited into the deep ocean^57^. Climate change affects predator–prey interactions, including virus–host interactions, and thereby global biogeochemical cycles^72^.

Oxygen minimum zones (OMZs) have expanded in the past 50 years as a result of ocean warming, which reduces oxygen solubility^73–75^. OMZs are global sinks for reactive nitrogen, and microbial production of N_2_ and N_2_O accounts for ~25–50% of nitrogen loss from the ocean to the atmosphere. Furthermore, OMZs are the largest pelagic methane reservoirs in the ocean and contribute substantially to open ocean methane cycling. The observed and predicted future expansion of OMZs may therefore considerably affect ocean nutrient and greenhouse gas budgets, and the distributions of oxygen-dependent organisms^73–75^.

The top 50 cm of deep-sea sediments contains ~1 × 10^29^ microorganisms^8,16^, and the total abundances of archaea and bacteria in these sediments increase with latitude (from 34° N to 79° N) with specific taxa (such as Marine Group I Thaumarchaeota) contributing disproportionately to the increase^76^. Benthic microorganisms show biogeographic patterns and respond to variations in the quantity and quality of the particulate matter sinking to the seafloor^77^. As a result, climate change is expected to particularly affect the functional processes that deep-sea benthic archaea perform (such as ammonia oxidation) and associated biogeochemical cycles^76^.

Aerosols affect cloud formation, thereby influencing sunlight irradiation and precipitation, but the extent to which and the manner in which they influence climate remains uncertain^78^. Marine aerosols consist of a complex mixture of sea salt, non-sea-salt sulfate and organic molecules and can function as nuclei for cloud condensation, influencing the radiation balance and, hence, climate^79,80^. For example, biogenic aerosols in remote marine environments (for example, the Southern Ocean) can increase the number and size of cloud droplets, having similar effects on climate as aerosols in highly polluted regions^80–83^. Specifically, phytoplankton emit dimethylsulfide, and its derivate sulfate promotes cloud condensation^79,84^. Understanding the ways in which marine phytoplankton contribute to aerosols will allow better predictions of how changing ocean conditions will affect clouds and feed back on climate^84^. In addition, the atmosphere itself contains ~10^22^ microbial cells, and determining the ability of atmospheric microorganisms to grow and form aggregates will be valuable for assessing their influence on climate^8^.

Vegetated coastal habitats are important for carbon sequestration, determined by the full trophic spectrum from predators to herbivores, to plants and their associated microbial communities^85^. Human activity, including anthropogenic climate change, has reduced these habitats over the past 50 years by 25–50%, and the abundance of marine predators has dropped by up to 90%^85–87^. Given such extensive perturbation, the effects on microbial communities need to be evaluated because microbial activity determines how much carbon is remineralized and released as CO_2_ and CH_4_.

### Climate change affects microorganisms

Climate change perturbs interactions between species and forces species to adapt, migrate and be replaced by others or go extinct^28,88^. Ocean warming, acidification, eutrophication and overuse (for example, fishing, tourism) together cause the decline of coral reefs and may cause ecosystems shifts towards macroalgae^89–93^ and benthic cyanobacterial mats^94,95^. The capacity for corals to adapt to climate change is strongly influenced by the responses of their associated microorganisms, including microalgal symbionts and bacteria^96–98^. The hundreds to thousands of microbial species that live on corals are crucial for host health, for example by recycling the waste products, by provisioning essential nutrients and vitamins and by assisting the immune system to fight pathogens^99^. However, environmental perturbation or coral bleaching can change the coral microbiome rapidly. Such shifts undoubtedly influence the ecological functions and stability of the coral–microorganism system, potentially affecting the capacity and pace at which corals adapt to climate change, and the relationships between corals and other components of the reef ecosystem^99,100^.

Generally, microorganisms can disperse more easily than macroscopic organisms. Nevertheless, biogeographic distinctions occur for many microbial species, with dispersal, lifestyle (for example, host association) and environmental factors strongly influencing community composition and function^54,101–103^. Ocean currents and thermal and latitudinal gradients are particularly important for marine communities^104,105^. If movement to more favourable environments is impossible, evolutionary change may be the only survival mechanism^88^. Microorganisms, such as bacteria, archaea and microalgae, with large population sizes and rapid asexual generation times have high adaptive potential^22^. Relatively few studies have examined evolutionary adaptation to ocean acidification or other climate change-relevant environmental variables^22,28^. Similarly, there is limited understanding of the molecular mechanisms of physiological responses and the implications of those responses for biogeochemical cycles^18^.

However, several studies have demonstrated effects of elevated CO_2_ levels on individual phytoplankton species, which may disrupt broader ecosystem-level processes. A field experiment demonstrated that increasing CO_2_ levels provide a selective advantage to a toxic microalga, *Vicicitus globosus*, leading to disruption of organic matter transfer across trophic levels^106^. The marine cyanobacterial genus *Trichodesmium* responds to long-term (4.5-year) exposure to elevated CO_2_ levels with irreversible genetic changes that increase nitrogen fixation and growth^107^. For the photosynthetic green alga *Ostreococcus tauri*, elevated CO_2_ levels increase growth, cell size and carbon-to-nitrogen ratios^108^. Higher CO_2_ levels also affect the population structure of *O. tauri*, with changes in ecotypes and niche occupation, thereby affecting the broader food webs and biogeochemical cycles^108^. Rather than producing larger cells, the calcifying phytoplankton species *Emiliania huxleyi* responds to the combined effects of elevated temperature and elevated CO_2_ levels (and associated acidification) by producing smaller cells that contain less carbon^109^. However, for this species, overall production rates do not change as a result of evolutionary adaptation to higher CO_2_ levels^109^. Responses to CO_2_ levels differ between communities (for example, between Arctic phytoplankton and Antarctic phytoplankton^110^). A mesocosm study identified variable changes in the diversity of viruses that infect *E. huxleyi* when it is growing under elevated CO_2_ levels, and noted the need to determine whether elevated CO_2_ levels directly affected viruses, hosts or the interactions between them^111^. These examples illustrate the need to improve our understanding of evolutionary processes and incorporate that knowledge into predictions of the effects of climate change.

Ocean acidification presents marine microorganisms with pH conditions well outside their recent historical range, which affects their intracellular pH homeostasis^18,112^. Species that are less adept at regulating internal pH will be more affected, and factors such as organism size, aggregation state, metabolic activity and growth rate influence the capacity for regulation^112^.

Lower pH causes bacteria and archaea to change gene expression in ways that support cell maintenance rather than growth^18^. In mesocosms with low phytoplankton biomass, bacteria committed more resources to pH homeostasis than bacteria in nutrient-enriched mesocosms with high phytoplankton biomass. Consequently, ocean acidification is predicted to alter the microbial food web via changes in cellular growth efficiency, carbon cycling and energy fluxes, with the biggest effects expected in the oligotrophic regions, which include most of the ocean^18^. Experimental comparisons of *Synechococcus* sp. growth under both present and predicted future pH concentrations showed effects not only on the cyanobacteria but also on the cyanophage viruses that infect them^113^.

Environmental temperature and latitude correlate with the diversity, distribution and/or temperature optimum (*T*_opt_) of certain marine taxa, with models predicting that rising temperatures will cause a poleward shift of cold-adapted communities^52,114–118^. However, *T*_opt_ of phytoplankton from polar and temperate waters was found to be substantially higher than environmental temperatures, and an eco-evolutionary model predicted that *T*_opt_ for tropical phytoplankton would be substantially higher than observed experimental values^116^. Understanding how well microorganisms are adapted to environmental temperature and predicting how they will respond to warming requires assessments of more than *T*_opt_, which is generally a poor indicator of physiological and ecological adaptation of microorganisms from cold environments^119^.

Many environmental and physiological factors influence the responses and overall competitiveness of microorganisms in their native environment. For example, elevated temperatures increase protein synthesis in eukaryotic phytoplankton while reducing cellular ribosome concentration^120^. As the biomass of eukaryotic phytoplankton is ~1 Gt C (ref.^13^) and ribosomes are phosphate rich, climate change-driven alteration of their nitrogen-to-phosphate ratio will affect resource allocation in the global ocean^120^. Ocean warming is thought to favour smaller plankton types over larger ones, changing biogeochemical fluxes such as particle export^121^. Increased ocean temperatures, acidification and decreased nutrient supplies are projected to increase the extracellular release of dissolved organic matter from phytoplankton, with changes in the microbial loop possibly causing increased microbial production at the expense of higher trophic levels^122^. Warming can also alleviate iron limitation of nitrogen-fixing cyanobacteria, with potentially profound implications for new nitrogen supplied to food webs of the future warming oceans^123^. Careful attention needs to be paid to how to quantify and interpret responses of environmental microorganisms to ecosystem changes and stresses linked to climate change^124,125^. Key questions thus remain about the functional consequences of community shifts, such as changes in carbon remineralization versus carbon sequestration, and nutrient cycling.

## Terrestrial biome

There is ~100-fold more terrestrial biomass than marine biomass, and terrestrial plants account for a large proportion of Earth’s total biomass^15^. Terrestrial plants perform roughly half of net global primary production^30,67^. Soils store ~2,000 billion tonnes of organic carbon, which is more than the combined pool of carbon in the atmosphere and vegetation^126^. The total number of microorganisms in terrestrial environments is ~10^29^, similar to the total number in marine environments^8^. Soil microorganisms regulate the amount of organic carbon stored in soil and released back to the atmosphere, and indirectly influence carbon storage in plants and soils through provision of macronutrients that regulate productivity (nitrogen and phosphorus)^126,127^. Plants provide a substantial amount of carbon to their mycorrhizal fungal symbionts, and in many ecosystems, mycorrhizal fungi are responsible for substantial amounts of nitrogen and phosphorus acquisition by plants^128^.

Plants remove CO_2_ from the atmosphere through photosynthesis and create organic matter that fuels terrestrial ecosystems. Conversely, autotrophic respiration by plants (60 Pg C per year) and heterotrophic respiration by microorganisms (60 Pg C per year) release CO_2_ back into the atmosphere^126,129^. Temperature influences the balance between these opposing processes and thus the capacity of the terrestrial biosphere to capture and store anthropogenic carbon emissions (currently, storing approximately one quarter of emissions) (Fig. 1). Warming is expected to accelerate carbon release into the atmosphere^129^.

Forests cover ~30% of the land surface, contain ~45% of terrestrial carbon, make up ~50% of terrestrial primary production and sequester up to 25% of anthropogenic CO_2_ (refs^130,131^). Grasslands cover ~29% of the terrestrial surface^132^. Non-forested, arid and semiarid regions (47%) are important for the carbon budget and respond differently to anthropogenic climate change than forested regions^132,133^. Lakes make up ~4% of the non-glaciated land area^134^, and shallow lakes emit substantial amounts of CH_4_ (refs^135,136^). Peat (decomposed plant litter) covers ~3% of the land surface and, due to plant productivity exceeding decomposition, intact peatlands function as a global carbon sink and contain ~30% of global soil carbon^137,138^. In permafrost, the accumulation of carbon in organic matter (remnants of plants, animals and microorganisms) far exceeds the respiratory losses, creating the largest terrestrial carbon sink^139–141^. Climate warming of 1.5–2 °C (relative to the global mean surface temperature in 1850–1900) is predicted to reduce permafrost by 28–53% (compared with levels in 1960–1990)^142^, thereby making large carbon reservoirs available for microbial respiration and greenhouse gas emissions.

Evaluations of the top 10 cm of soil^143^ and whole-soil profiles to 100 cm deep, which contain older stocks of carbon^144^, demonstrate that warming increases carbon loss to the atmosphere. Explaining differences in carbon loss between different soil sites will require a greater range of predictive variables (in addition to soil organic matter content, temperature, precipitation, pH and clay content)^145,146^. Nevertheless, predictions from global assessments of responses to warming indicate that terrestrial carbon loss under warming is causing a positive feedback that will accelerate the rate of climate change^143^, particularly in cold and temperate soils, which store much of the global soil carbon^147^.

### Microorganisms affect climate change

Higher CO_2_ levels in the atmosphere increase primary productivity and thus forest leaf and root litter^148–150^, which leads to higher carbon emissions due to microbial degradation^151^. Higher temperatures promote higher rates of terrestrial organic matter decomposition^152^. The effect of temperature is not just a kinetic effect on microbial reaction rates but results from plant inputs stimulating microbial growth^152–154^.

Several local environmental factors (such as microbial community composition, density of dead wood, nitrogen availability and moisture) influence rates of microbial activity (for example, fungal colonization of wood) necessitating Earth system model predictions of soil carbon losses through climate warming to incorporate local controls on ecosystem processes^155^. In this regard, plant nutrient availability affects the net carbon balance in forests, with nutrient-poor forests releasing more carbon than nutrient-rich forests^156^. Microbial respiration may be lower in nutrient-rich forests as plants provide less carbon (for example, as root exudates) to rhizosphere microorganisms^157^.

Plants release ~50% of fixed carbon into the soil, which is available for microbial growth^158–160^. In addition to microorganisms using exudates as an energy source, exudates can disrupt mineral–organic associations, liberating organic compounds from minerals that are used for microbial respiration, thereby increasing carbon release^159^. The relevance of these plant–mineral interactions illustrates the importance of biotic–abiotic interactions, in addition to biotic interactions (plant–microorganism) when one is evaluating the influence of climate change^159^. Thermodynamic models that incorporate the interactions of microorganisms and secreted enzymes with organic matter and minerals have been used to predict soil carbon–climate feedbacks in response to increasing temperature; one study predicted more variable but weaker soil carbon–climate feedbacks from a thermodynamic model than from static models^160^.

The availability of soil organic matter for microbial degradation versus long-term storage depends on many environmental factors, including the soil mineral characteristics, acidity and redox state; water availability; climate; and the types of microorganisms present in the soil^161^. The nature of the organic matter, in particular substrate complexity, affects microbial decomposition. Furthermore, the microbial capacity to access organic matter differs between soil types (for example, with different clay content)^162^. If access is taken into account, increasing atmospheric CO_2_ levels are predicted to allow greater microbial decomposition and less soil retention of organic carbon^162^.

Elevated CO_2_ concentrations enhance competition for nitrogen between plants and microorganisms^163^. Herbivores (invertebrates and mammals) affect the amount of organic matter that is returned to soil and thereby microbial biomass and activity^164^. For example, grasshoppers diminish plant biomass and plant nitrogen demand, thereby increasing microbial activity^163^. Climate change can reduce herbivory, resulting in overall alterations to global nitrogen and carbon cycles that reduce terrestrial carbon sequestration^163^. Detritivores (for example, earthworms) influence greenhouse gas emissions by indirectly affecting plants (for example, by increasing soil fertility) and soil microorganisms^165^. Earthworms modify soils through feeding, burrowing and deposition of waste products. The anaerobic gut environment of earthworms harbours microorganisms that perform denitrification and produce N_2_O. Earthworms enhance soil fertility, and their presence can result in net greenhouse gas emissions^165^, although the combined effects of increased temperature and decreased rainfall on detritivore feeding and microbial respiration may reduce emissions^166^.

In peatlands, decay-resistant litter (for example, antimicrobial phenolics and polysaccharides of *Sphagnum* mosses) inhibits microbial decomposition, and water saturation restricts oxygen exchange and promotes the growth of anaerobes and release of CO_2_ and CH_4_ (refs^137,167^). Increased temperature and reduced soil water content caused by climate change promote the growth of vascular plants (ericaceous shrubs) but reduce the productivity of peat moss. Changes in plant litter composition and associated microbial processes (for example, reduced immobilization of nitrogen and enhanced heterotrophic respiration) are switching peatlands from carbon sinks to carbon sources^137^.

Melting and degradation of permafrost allows microbial decomposition of previously frozen carbon, releasing CO_2_ and CH_4_ (refs^139–141,168,169^). Coastal permafrost erosion will lead to the mobilization of large quantities of carbon to the ocean, with potentially large CO_2_ emissions occurring through increased microbial remineralization^170^, causing a positive feedback loop that accelerates climate change^139–141,168–171^. Melting of permafrost leads to increases in water-saturated soils^172^, which promotes anaerobic CH_4_ production by methanogens and CO_2_ production by a range of microorganisms. Production is slow compared with metabolism in drained aerobic soils, which release CO_2_ rather than CH_4_. However, a 7-year laboratory study of CO_2_ and CH_4_ production found that once methanogen communities became active in thawing permafrost, equal amounts of CO_2_ and CH_4_ were formed under anoxic conditions, and it was predicted that by the end of the century, carbon emissions from anoxic environments will drive climate change to a greater extent than emissions from oxic environments^172^.

A 15-year mesocosm study that simulated freshwater lake environments determined that the combined effects of eutrophication and warming can lead to large increases in CH_4_ ebullition (bubbles from accumulated gas)^135^. As small lakes are susceptible to eutrophication and tend to be located in climate-sensitive regions, the role of lake microorganisms in contributing to global greenhouse gas emissions needs to be evaluated^135,136^.

### Climate change affects microorganisms

Shifts in climate can influence the structure and diversity of microbial communities directly (for example, seasonality and temperature) or indirectly (for example, plant composition, plant litter and root exudates). Soil microbial diversity influences plant diversity and is important for ecosystem functions, including carbon cycling^173,174^.

Both short-term laboratory warming and long-term (more than 50 years) natural geothermal warming initially increased the growth and respiration of soil microorganisms, leading to net CO_2_ release and subsequent depletion of substrates, causing a decrease in biomass and reduced microbial activity^175^. This implies that microbial communities do not readily adapt to higher temperatures, and the resulting effects on reaction rates and substrate depletion reduce overall carbon loss^175^. By contrast, a 10-year study found that soil communities adapted to increased temperature by changing composition and patterns of substrate use, leading to less carbon loss than would have occurred without adaptation^176^. Substantial changes in bacterial and fungal communities were also found in forest soils with a more than 20 °C average annual temperature range^177^, and in response to warming across a 9-year study of tall-grass prairie soils^178^.

Two studies assessed the effects of elevated temperatures on microbial respiration rates and mechanisms and outcomes of adaptation^179,180^. The studies examined a wide range of environmental temperatures (−2 to 28 °C), dryland soils (110 samples) and boreal, temperate and tropical soils (22 samples), and evaluated how communities respond to three different temperatures (~10–30 °C). Thermal adaptation was linked to biophysical characteristics of cell membranes and enzymes (reflecting activity-stability trade-offs^180^) and the genomic potential of microorganisms (with warmer environments having microbial communities with more diverse lifestyles^179^). Respiration rates per unit biomass were lower in soils from higher-temperature environments, indicating that thermal adaptation of microbial communities may lessen positive climate feedbacks. However, as respiration depends on multiple interrelated factors (not just on one variable, such as temperature), such mechanistic insights into microbial physiology need to be represented in biogeochemical models of possible positive climate feedbacks.

Microbial growth responses to temperature change are complex and varied^181^. Microbial growth efficiency is a measure of how effectively microorganisms convert organic matter into biomass, with lower efficiency meaning more carbon is released to the atmosphere^182,183^. A 1-week laboratory study found that increasing temperature led to increases in microbial turnover but no change in microbial growth efficiency, and predicted that warming would promote carbon accumulation in soil^183^. A field study spanning 18 years found microbial efficiency was reduced at higher soil temperature, with decomposition of recalcitrant, complex substrates increasing by the end of the period along with a net loss of soil carbon^182^.

Similarly, in a 26-year forest-soil warming study, temporal variation occurred in organic matter decomposition and CO_2_ release^184^, leading to changes in microbial community composition and carbon use efficiency, reduced microbial biomass and reduced microbially accessible carbon^184^. Overall, the study predicted anthropogenic climate change to cause long-term, increasing and sustained carbon release^184^. Similar predictions arise from Earth system models that simulate microbial physiological responses^185^ or incorporate the effects of freezing and thawing of cold-climate soils^186^.

Climate change directly and indirectly influences microbial communities and their functions through several interrelated factors, such as temperature, precipitation, soil properties and plant input. As soil microorganisms in deserts are carbon limited, increased carbon input from plants promotes transformation of nitrogenous compounds, microbial biomass, diversity (for example, of fungi), enzymatic activity and use of recalcitrant organic matter^133^. Although these changes may enhance respiration and net loss of carbon from soil, the specific characteristics of arid and semiarid regions may mean they could function as carbon sinks^133^. However, a study of 19 temperate grassland sites found that seasonal differences in rainfall constrain biomass accumulation^132^. To better understand aboveground plant-biomass responses to CO_2_ levels and seasonal precipitation, we also need improved knowledge of microbial community responses and functions.

Metagenome data, including metagenome-assembled genomes, provide knowledge of key microbial groups that metabolize organic matter and release CO_2_ and CH_4_ and link these groups to the biogeochemistry occurring in thawing permafrost^187–191^. Tundra microbial communities change in the soil layer of permafrost after warming^192^. Within 1.5 years of warming, the functional potential of the microbial communities changed markedly, with an increasing abundance of genes involved in aerobic and anaerobic carbon decomposition and nutrient cycling. Although microbial metabolism stimulates primary productivity by plants, the balance between microbial respiration and primary productivity results in a net release of carbon to the atmosphere^192^. When forests expand into warming regions of tundra, plant growth can produce a net loss of carbon, possibly as a result of root exudates stimulating microbial decomposition of native soil carbon^153,193^. Although there are reports of carbon accumulating owing to warming (for example, ref.^183^), most studies describe microbial community responses that result in carbon loss.

Rapid warming of the Antarctic Peninsula and associated islands resulted in range expansion of Antarctic hair grass (*Deschampsia antarctica*), as it outcompetes other indigenous species (for example, the moss *Sanionia uncinata*) through the superior capacity of its roots to acquire peptides and thus nitrogen^194^. The ability of the grass to be competitive depends on microbial digestion of extracellular proteins and generation of amino acids, nitrate and ammonium^194^. As warmer soils in this region harbour greater fungal diversity, climate change is predicted to cause changes in the fungal communities that will affect nutrient cycling and primary productivity^195^. Cyanobacterial diversity and toxin production within benthic mats from both the Antarctic Peninsula and the Arctic increased during 6 months of exposure to high growth temperatures^196^. A shift to toxin-producing species or increased toxin production by existing species could affect polar freshwater lakes, where cyanobacteria are often the dominant benthic primary producers^196^.

Climate change is likely to increase the frequency, intensity and duration of cyanobacterial blooms in many eutrophic lakes, reservoirs and estuaries^197,198^. Bloom-forming cyanobacteria produce a variety of neurotoxins, hepatotoxins and dermatoxins, which can be fatal to birds and mammals (including waterfowl, cattle and dogs) and threaten the use of waters for recreation, drinking water production, agricultural irrigation and fisheries^198^. Toxic cyanobacteria have caused major water quality problems, for example in Lake Taihu (China), Lake Erie (USA), Lake Okeechobee (USA), Lake Victoria (Africa) and the Baltic Sea^198–200^.

Climate change favours cyanobacterial blooms both directly and indirectly^198^. Many bloom-forming cyanobacteria can grow at relatively high temperatures^201^. Increased thermal stratification of lakes and reservoirs enables buoyant cyanobacteria to float upwards and form dense surface blooms, which gives them better access to light and hence a selective advantage over nonbuoyant phytoplankton organisms^202,203^. Protracted droughts during summer increase water residence times in reservoirs, rivers and estuaries, and these stagnant warm waters can provide ideal conditions for cyanobacterial bloom development^204^.

The capacity of the harmful cyanobacterial genus *Microcystis* to adapt to elevated CO_2_ levels was demonstrated in both laboratory and field experiments^205^. *Microcystis* spp. take up CO_2_ and HCO_3_^−^ and accumulate inorganic carbon in carboxysomes, and strain competitiveness was found to depend on the concentration of inorganic carbon. As a result, climate change and increased CO_2_ levels are expected to affect the strain composition of cyanobacterial blooms^205^.

## Agriculture

According to the World Bank (World Bank data on agricultural land), nearly 40% of the terrestrial environment is devoted to agriculture. This proportion is predicted to increase, leading to substantial changes in soil cycling of carbon, nitrogen and phosphorus, among other nutrients. Furthermore, these changes are associated with a marked loss of biodiversity^206^, including of microorganisms^207^. There is increasing interest in using plant-associated and animal-associated microorganisms to increase agricultural sustainability and mitigate the effects of climate change on food production, but doing so requires a better understanding of how climate change will affect microorganisms.

### Microorganisms affect climate change

**Fig. 2:**
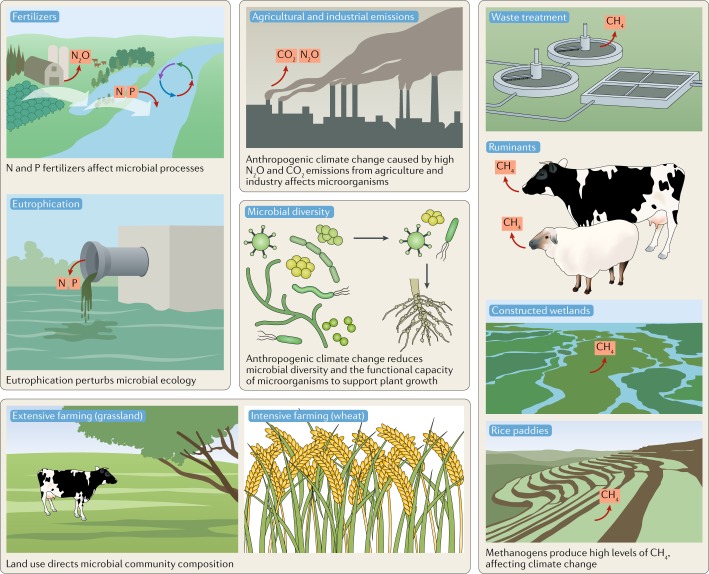
Agriculture and other human activities that affect microorganisms. Agricultural practices influence microbial communities in specific ways. Land usage (for example, plant type) and sources of pollution (for example, fertilizers) perturb microbial community composition and function, thereby altering natural cycles of carbon, nitrogen and phosphorus transformations. Methanogens produce substantial quantities of methane directly from ruminant animals (for example, cattle, sheep and goats) and saturated soils with anaerobic conditions (for example, rice paddies and constructed wetlands). Human activities that cause a reduction in microbial diversity also reduce the capacity for microorganisms to support plant growth.

Methanogens produce methane in natural and artificial anaerobic environments (sediments, water-saturated soils such as rice paddies, gastrointestinal tracts of animals (particularly ruminants), wastewater facilities and biogas facilities), in addition to the anthropogenic methane production associated with fossil fuels^208^ (Fig. 2). The main sinks for CH_4_ are atmospheric oxidation and microbial oxidation in soils, sediments and water^208^. Atmospheric CH_4_ levels have risen sharply in recent years (2014–2017) but the reasons are unclear so far, although they involve increased emissions from methanogens and/or fossil fuel industries and/or reduced atmospheric CH_4_ oxidation, thereby posing a major threat to controlling climate warming^209^.

Rice feeds half of the global population^210^, and rice paddies contribute ~20% of agricultural CH_4_ emissions despite covering only ~10% of arable land. Anthropogenic climate change is predicted to double CH_4_ emissions from rice production by the end of the century^210^. Ruminant animals are the largest single source of anthropogenic CH_4_ emissions, with a 19–48 times larger carbon footprint for ruminant meat production than plant-based high-protein foods^211^. Even the production of meat from non-ruminant animals (such as pigs, poultry and fish) produces 3–10 times more CH_4_ than high-protein plant foods^211^.

The combustion of fossil fuels and the use of fertilizers has greatly increased the environmental availability of nitrogen, perturbing global biogeochemical processes and threatening ecosystem sustainability^212,213^. Agriculture is the largest emitter of the potent greenhouse gas N_2_O, which is released by microbial oxidation and reduction of nitrogen^214^. The enzyme N_2_O reductase in rhizobacteria (in root nodules) and other soil microorganisms can also convert N_2_O to N_2_ (not a greenhouse gas). Climate change perturbs the rate at which microbial nitrogen transformations occur (decomposition, mineralization, nitrification, denitrification and fixation) and release N_2_O (ref.^213^). There is an urgent need to learn about the effects of climate change and other human activities on microbial transformations of nitrogen compounds.

### Climate change affects microorganisms

Crop farming ranges from extensively managed (small inputs of labour, fertilizer and capital) to intensively managed (large inputs). Increasing temperature and drought strongly affect the ability to grow crops^215^. Fungal-based soil food webs are common in extensively managed farming (for example, grasslands) and are better able to adapt to drought than bacterial-based food webs, which are common in intensive systems (for example, wheat)^216,217^. A global assessment of topsoil found that soil fungi and bacteria occupy specific niches and respond differently to precipitation and soil pH, indicating that climate change would have differential impacts on their abundance, diversity and functions^218^. Aridity, which is predicted to increase owing to climate change, reduces bacterial and fungal diversity and abundance in global drylands^219^. Reducing soil microbial diversity reduces the overall functional potential of microbial communities, thereby limiting their capacity to support plant growth^173^.

The combined effects of climate change and eutrophication caused by fertilizers can have major, potentially unpredictable effects on microbial competitiveness. For example, nutrient enrichment typically favours harmful algal blooms, but a different outcome was observed in the relatively deep Lake Zurich^220^. Reducing phosphorus inputs from fertilizers reduced eukaryotic phytoplankton blooms but increased the nitrogen-to-phosphorus ratio and thus the non-nitrogen-fixing cyanobacterium *Planktothrix rubescens* became dominant^220^. In the absence of effective predation, annual mixing has an important role in controlling cyanobacterial populations. However, warming increased thermal stratification and reduced mixing, thereby facilitating the persistence of the toxic cyanobacteria^220^.

## Infectious diseases

**Fig. 3:**
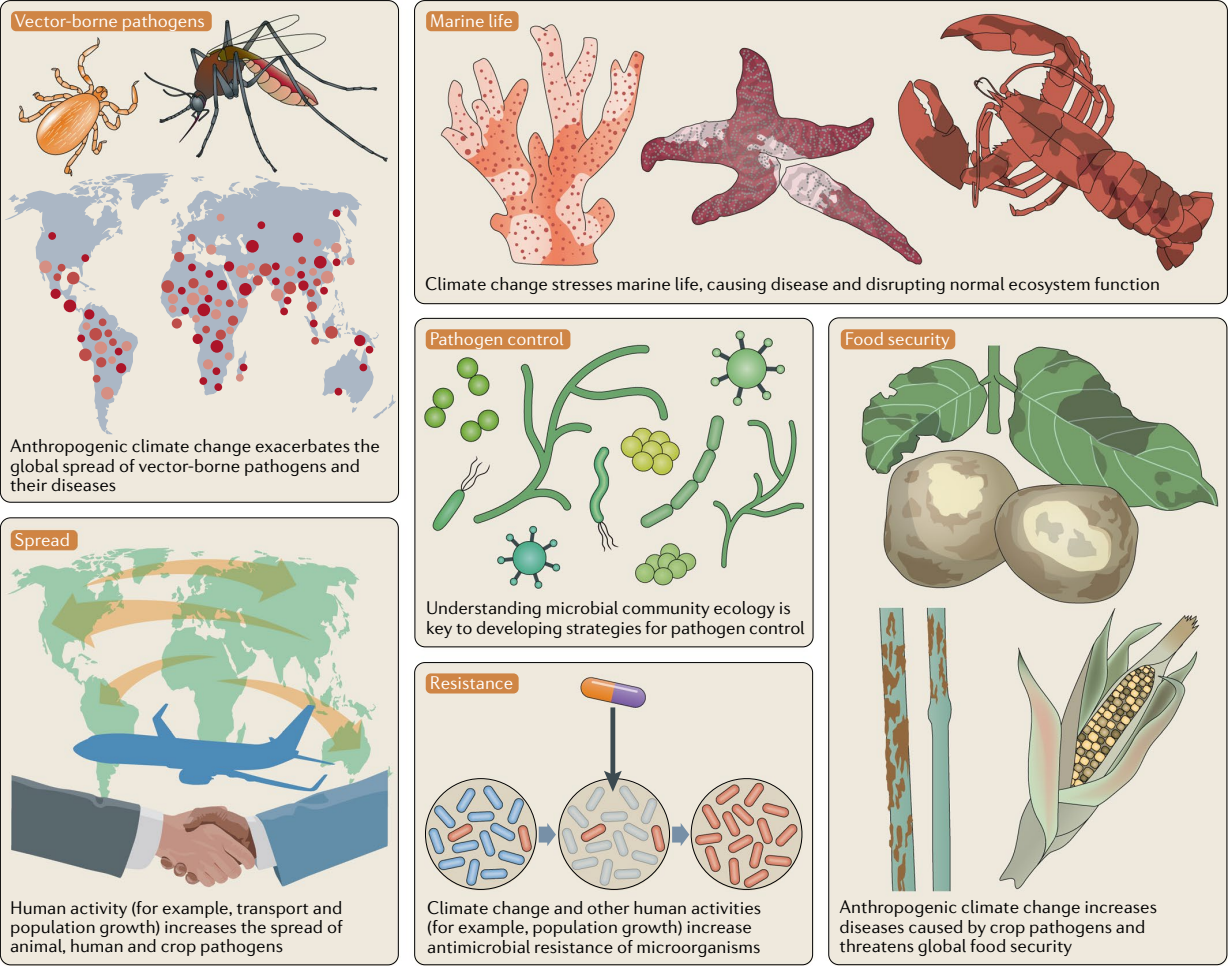
Climate change exacerbates the impact of pathogens. Anthropogenic climate change stresses native life, thereby enabling pathogens to increasingly cause disease. The impact on aquaculture, food-producing animals and crops threatens global food supply. Human activities, such as population growth and transport, combined with climate change increase antibiotic resistance of pathogens and the spread of waterborne and vector-borne pathogens, thereby increasing diseases of humans, other animals and plants.

Climate change affects the occurrence and spread of diseases in marine and terrestrial biota^221^ (Fig. 3), depending on diverse socioeconomic, environmental and host–pathogen-specific factors^222^. Understanding the spread of disease and designing effective control strategies requires knowledge of the ecology of pathogens, their vectors and their hosts, and the influence of dispersal and environmental factors^223^ (Table 1). For example, there is a strong link between increasing sea surface temperatures and coral disease and, although the disease mechanisms are not absolutely clear for all the different syndromes, associations with microbial pathogens exist^224–226^. Peaks in disease prevalence coincide with periodicities in the El Niño Southern Oscillation (ENSO)^227^. In particular, in some coral species, ocean warming can alter the coral microbiome, disrupting the host–symbiont equilibrium, shifting defensive mechanisms and nutrient cycling pathways that may contribute to bleaching and disease^99^. Ocean acidification may also directly cause tissue damage in organisms such as fish, potentially contributing to a weakened immune system that creates opportunities for bacterial invasion^228^.

**Table 1:** Transmission response of pathogens to climatic and environmental factors

Example pathogens or diseases	Climatic and environmental factors	Transmission parameters
***Vector-borne***		
West Nile virus	Precipitation, relative humidity, temperature, El Niño Southern Oscillation	Vector abundance, longevity and biting rate, pathogen replication rate in vector^273–276^
Malaria		
Dengue fever		
Lyme disease		
***Waterborne***		
Cholera	Temperature, precipitation variability, salinity, El Niño Southern Oscillation	Pathogen survival, pathogen replication in environment, pathogen transport^244,277–279^
Non-cholera *Vibrio* spp.		
*Cryptosporidium* spp.		
Rotavirus		
***Airborne***		
Influenza	Relative humidity, temperature, wind	Pathogen survival, pathogen and/or host dispersal^280–284^
Hantavirus		
Coccidioidomycosis		
***Foodborne***		
*Salmonella* spp.	Temperature, precipitation	Pathogen replication, human behaviour^239,240^
*Campylobacter* spp.		

Sea star species declined by 80–100% along an ~3000 km section of the North American west coast, with peak declines occurring during anomalous increases in sea surface temperatures^229^. As sea stars are important predators of sea urchins, loss of predation can cause a trophic cascade that affects kelp forests and associated marine biodiversity^229,230^. Given the effects of ocean warming on pathogen impacts, temperature monitoring systems have been developed for a wide range of marine organisms, including corals, sponges, oysters, lobsters and other crustaceans, sea stars, fish and sea grasses^231^.

Forest die-off caused by drought and heat stress can be exacerbated by pathogens^232^. For crops, a variety of interacting factors are important when one is considering response to pathogens, including CO_2_ levels, climatic changes, plant health and species-specific plant–pathogen interactions^233^. A broad range of microorganisms cause plant diseases (fungi, bacteria, viruses, viroids and oomycetes) and can, therefore, affect crop production, cause famines (for example, the oomycete *Phytophthora infestans* caused the Irish potato famine) and threaten food security^233^. An assessment of more than 600 crop pests (nematodes and insects) and pathogens since 1960 found an expansion towards the poles that is attributable to climate change^233^. The spread of pathogens and the emergence of disease are facilitated by transport and introduction of species and are influenced by effects of weather on dispersal and environmental conditions for growth^233^.

Climate change can increase the disease risk by altering host and parasite acclimation^234^. For ectotherms (such as amphibians), temperature can increase susceptibility to infection, possibly through perturbation of immune responses^234,235^. Monthly and daily unpredictable environmental temperature fluctuations increase the susceptibility of the Cuban tree frog to the pathogenic chytrid fungus *Batrachochytrium dendrobatidis*. The effect of increasing temperature on infection contrasts with decreased growth capacity of the fungus in pure culture, illustrating the importance of assessing host–pathogen responses (rather than extrapolating from growth rate studies of isolated microorganisms) when evaluating the relevance of climate change^234^.

Climate change is predicted to increase the rate of antibiotic resistance of some human pathogens^236^. Data from 2013–2015 suggest that an increase of the daily minimum temperature by 10 °C (which is conceivable for some parts of the USA by the end of the century) will lead to an increase in antibiotic resistance rates of *Escherichia coli*, *Klebsiella pneumoniae* and *Staphylococcus aureus* by 2–4% (up to 10% for certain antibiotics)^236^. Potential underlying mechanisms include elevated temperatures facilitating horizontal gene transfer of mobile genetic elements of resistance, and increased pathogen growth rates promoting environmental persistence, carriage and transmission^236^. Population growth, which amplifies climate change, is also an important factor in contributing to the development of resistance^236^.

Vector-borne, foodborne, airborne, waterborne and other environmental pathogens may be particularly susceptible to the effects of climate change^237–240^ (Table 1). For vector-borne diseases, climate change will affect the distribution of vectors and hence the range over which diseases are transmitted, as well as the efficiency with which vectors transmit pathogens. Efficiency depends on the time between a vector feeding on an infected host and the vector becoming infectious itself. At warmer temperatures, this time can be reduced substantially, providing more opportunity for transmission within the vector’s lifespan. Certain vector-borne diseases, such as bluetongue, an economically important viral disease of livestock, have already emerged in Europe in response to climate change, and larger, more frequent outbreaks are predicted to occur in the future^241^. For certain waterborne infections by pathogenic *Vibrio* spp., poleward spread correlates with increasing global temperature and lower salinity of aquatic environments in coastal regions (such as estuaries) caused by increased precipitation^242^. These changed conditions can promote the growth of *Vibrio* spp. in the environment^242^. Increasing sea surface temperatures also correlate with increases in *Vibrio cholerae* infections in Bangladesh^243^, infections with several human-pathogenic *Vibrio* spp. in the Baltic Sea region^242^ and the abundance of *Vibrio* spp. (including human pathogens) in the North Atlantic and North Sea^244^.

Malaria and dengue fever are two vector-borne diseases that are known to be highly sensitive to climate conditions, and thus their spatial distributions are expected to shift in response to climate change^4,141,245^. Climate change can facilitate the spread of vector-borne pathogens by prolonging the transmission season, increasing the rate of replication of pathogens in the vector and increasing the number and geographic range of mosquitoes. This is especially the case for *Aedes aegypti*, the major vector of dengue, Zika, chikungunya and yellow fever viruses, which is currently limited to tropical and subtropical regions because it cannot survive cold winters. In combination with other mosquito-borne diseases (such as West Nile fever and Japanese encephalitis) and tick-borne diseases (such as Lyme disease), millions of people are predicted to be newly at risk under climate change^4,238,246–249^.

Many infectious diseases, including several vector-borne and waterborne diseases, are strongly influenced by climate variability caused by large-scale climate phenomena such as the ENSO, which disrupts normal rainfall patterns and changes temperatures in about two thirds of the globe every few years. Associations with ENSO have been reported for malaria, dengue fever, Zika virus disease, cholera, plague, African horse sickness and many other important human and animal diseases^250–254^.

Adaptation of species to their local environment has been studied less in microorganisms than in animals (including humans) and plants, although the mechanisms and consequences of adaptation have been studied in natural and experimental microbial populations^255^. Viral, bacterial and fungal pathogens of plants and animals (such as crops, humans and livestock) adapt to abiotic and biotic factors (such as temperature, pesticides, interactions between microorganisms and host resistance) in ways that affect ecosystem function, human health and food security^255^. The cyclic feedback between microbial response and human activity is well illustrated by the adaptation patterns of pathogenic agricultural fungi^256^. Because agricultural ecosystems have common global features (for example, irrigation, fertilizer use and plant cultivars) and human travel and transport of plant material readily disperse crop pathogens, ‘agro-adapted’ pathogens have a higher potential to cause epidemics and pose a greater threat to crop production than naturally occurring strains^256^. The ability of fungal pathogens to expand their range and invade new habitats by evolving to tolerate higher temperatures compounds the threat fungal pathogens pose to both natural and agricultural ecosystems^257^.

## Microbial mitigation of climate change

An improved understanding of microbial interactions would help underpin the design of measures to mitigate and control climate change and its effects (see also ref.^7^). For example, understanding how mosquitoes respond to the bacterium *Wolbachia* (a common symbiont of arthropods) has resulted in a reduction of the transmission of Zika, dengue and chikungunya viruses through the introduction of *Wolbachia* into populations of *A. aegypti* mosquitoes and releasing them into the environment^258^. In agriculture, progress in understanding the ecophysiology of microorganisms that reduce N_2_O to harmless N_2_ provides options for mitigating emissions^214,259^. The use of bacterial strains with higher N_2_O reductase activity has lowered N_2_O emissions from soybean, and both natural and genetically modified strains with higher N_2_O reductase activity provide avenues for mitigating N_2_O emissions^214^. Manipulating the rumen microbiota^260^ and breeding programmes that target host genetic factors that change microbial community responses^261^ are possibilities for reducing methane emission from cattle. In this latter case, the aim would be to produce cattle lines that sustain microbial communities producing less methane without affecting the health and productivity of the animals^261^. Fungal proteins can replace meat, lowering dietary carbon footprints^262^.

Biochar is an example of an agricultural solution for broadly and indirectly mitigating microbial effects of climate change. Biochar is produced from thermochemical conversion of biomass under oxygen limitation and improves the stabilization and accumulation of organic matter in iron-rich soils^263^. Biochar improves organic matter retention by reducing microbial mineralization and reducing the effect of root exudates on releasing organic material from minerals, thereby promoting growth of grasses and reducing the release of carbon^263^.

A potentially large-scale approach to mitigation is the use of constructed wetlands to generate cellulosic biofuel using waste nitrogen from wastewater treatment; if all waste in China were used, it could supply the equivalent of 7% of China’s gasoline consumption^264^. Such major developments of constructed wetlands would require the characterization and optimization of their core microbial consortia to manage their emissions of greenhouse gases and optimize environmental benefits^265^.

Microbial biotechnology can provide solutions for sustainable development^266^, including in the provision (for example, of food) and regulation (for example, of disease or of emissions and capture of greenhouse gases) of ecosystem services for humans, animals and plants. Microbial technologies provide practical solutions (chemicals, materials, energy and remediation) for achieving many of the 17 United Nations Sustainable Development Goals, addressing poverty, hunger, health, clean water, clean energy, economic growth, industry innovation, sustainable cities, responsible consumption, climate action, life below water, and life on land^6^ (Box 1). Galvanizing support for such actions will undoubtedly be facilitated by improving public understanding of the key roles of microorganisms in global warming, that is, through attainment of microbiology literacy in society^7^.

## Conclusion

Microorganisms make a major contribution to carbon sequestration, particularly marine phytoplankton, which fix as much net CO_2_ as terrestrial plants. For this reason, environmental changes that affect marine microbial photosynthesis and subsequent storage of fixed carbon in deep waters are of major importance for the global carbon cycle. Microorganisms also contribute substantially to greenhouse gas emissions via heterotrophic respiration (CO_2_), methanogenesis (CH_4_) and denitrification (N_2_O).

Many factors influence the balance of microbial greenhouse gas capture versus emission, including the biome, the local environment, food web interactions and responses, and particularly anthropogenic climate change and other human activities (Figs 1–3).

Human activity that directly affects microorganisms includes greenhouse gas emissions (particularly CO_2_, CH_4_ and N_2_O), pollution (particularly eutrophication), agriculture (particularly land usage) and population growth, which positively feeds back on climate change, pollution, agricultural practice and the spread of disease. Human activity that alters the ratio of carbon uptake relative to release will drive positive feedbacks and accelerate the rate of climate change. By contrast, microorganisms also offer important opportunities for remedying human-caused problems through improved agricultural outcomes, production of biofuels and remediation of pollution.

Addressing specific issues involving microorganisms will require targeted laboratory studies of model microorganisms (Box 2). Laboratory probing of microbial responses should assess environmentally relevant conditions, adopt a ‘microbcentric’ view of environmental stressors and be followed up by field tests. Mesocosm and in situ field experiments are particularly important for gaining insight into community-level responses to real environmental conditions. Effective experimental design requires informed decision-making, involving knowledge from multiple disciplines specific to marine (for example, physical oceanography) and terrestrial (for example, geochemistry) biomes.

To understand how microbial diversity and activity that govern small-scale interactions translate to large system fluxes, it will be important to scale findings from individuals to communities and to whole ecosystems. Earth system modellers need to include microbial contributions that account for physiological and adaptive (evolutionary) responses to biotic (including other microorganisms, plants and organic matter substrates) and abiotic (including mineral surfaces, ocean physics and chemistry) forcings.

We must improve our quantitative understanding of the global marine and soil microbiome. To understand biogeochemical cycling and climate change feedbacks at any location around the world, we need quantitative information about the organisms that drive elemental cycling (including humans, plants and microorganisms), and the environmental conditions (including climate, soil physiochemical characteristics, topography, ocean temperature, light and mixing) that regulate the activity of those organisms. The framework for quantitative models exists, but to a large extent these models lack mechanistic details of marine and terrestrial microorganisms. The reason for this omission has less to do with how to construct such a model mathematically but instead stems from the paucity of physiological and evolutionary data allowing robust predictions of microbial responses to environmental change. A focused investment into expanding this mechanistic knowledge represents a critical path towards generating the global models essential for benchmarking, scaling and parameterizing Earth system model predictions of current and future climate.

Extant life has evolved over billions of years to generate vast biodiversity, and microbial biodiversity is practically limitless compared with macroscopic life. Biodiversity of macroscopic organisms is rapidly declining because of human activity, suggesting that the biodiversity of host-specific microorganisms of animal and plant species will also decrease. However, compared with macroscopic organisms, we know far less about the connections between microorganisms and anthropogenic climate change. We can recognize the effects of microorganisms on climate change and climate change on microorganisms, but what we have learned is incomplete, complex and challenging to interpret. It is therefore not surprising that challenges exist for defining causes and effects of anthropogenic climate change on biological systems. Nevertheless, there is no doubt that human activity is causing climate change, and this is perturbing normal ecosystem function around the globe (Box 1). Across marine and terrestrial biomes, microbially driven greenhouse gas emissions are increasing and positively feeding back on climate change. Irrespective of the fine details, the microbial compass points to the need to act (Box 2). Ignorance of the role of, effects on and feedback response of microbial communities to climate change can lead to our own peril. An immediate, sustained and concerted effort is required to explicitly include microorganisms in research, technology development, and policy and management decisions. Microorganisms not only contribute to the rate of climate change but can also contribute immensely to its effective mitigation and our adaptation tools.

Box 2 A call to action The microbiologists’ warning calls for:

- Greater recognition that all multicellular organisms, including humans, rely on microorganisms for their health and functioning; microbial life is the support system of the biosphere.
- The inclusion of microorganisms in mainstream climate change research, particularly research addressing carbon and nitrogen fluxes.
- Experimental design that accounts for environmental variables and stresses (biotic and abiotic) that are relevant to the microbial ecosystem and climate change responses.
- Investigation of the physiological, community and evolutionary microbial responses and feedbacks to climate change.
- A focus on microbial feedback mechanisms in the monitoring of greenhouse gas fluxes from marine and terrestrial biomes and agricultural, industrial, waste and health sectors and investment in long-term monitoring.
- Incorporation of microbial processes into ecosystem and Earth system models to improve predictions under climate change scenarios.
- The development of innovative microbial technologies to minimize and mitigate climate change impacts, reduce pollution and eliminate reliance on fossil fuels.
- The introduction of teaching of personally, societally, environmentally and sustainability relevant aspects of microbiology in school curricula, with subsequent upscaling of microbiology education at tertiary levels, to achieve a more educated public and appropriately trained scientists and workforce.
- Explicit consideration of microorganisms for the development of policy and management decisions.
- A recognition that all key biosphere processes rely on microorganisms and are greatly affected by human behaviour, necessitating integration of microbiology in the management and advancement of the United Nations Sustainable Development Goals.

## Glossary

Habitats: Environments in which an organism normally lives; for example, lake, forest, sediment and polar environments represent distinct types of habitats.
Ecosystem: The interacting community of organisms and non-living components such as minerals, nutrients, water, weather and topographic features present in a specific environment.
Food web: Interconnecting components describing the trophic (feeding) interactions in an ecosystem, often consisting of multiple food chains; for example, marine microbial primary producers and heterotrophic remineralizers through to the highest trophic predators or trees as primary producers, herbivores and microbial nitrogen fixers and remineralizers.
Subsurface: The area below Earth’s surface, with subsurface ecosystems extending down for several kilometres and including terrestrial deep aquifer, hydrocarbon and mine systems, and marine sediments and the ocean crust.
Eutrophication: Increased input of minerals and nutrients to an aquatic system; typically nitrogen and phosphorus input from fertilizers, sewage and detergents.
Phytoplankton: Single-celled, chlorophyll-containing microorganisms (eukaryotes and bacteria) that grow photosynthetically and drift relatively passively with the current in oceans or lakes.
Biomes: Systems containing multiple ecosystems that have common physical properties (such as climate and geology); here ‘biome’ is used to refer to all terrestrial environments (continents) and all marine environments (seas and oceans).
Phototrophic: Using sunlight to generate energy for growth.
Water column: The water layer in a lake or ocean.
Stratification: Water layers forming due to a difference in the density of water between the surface and deeper waters; stratification is increasing owing to warming of surface waters and freshwater input from precipitation and ice melting.
Remineralizing: Converting organic matter back into its constituent inorganic components; remineralization by marine and terrestrial heterotrophs involves respiration that releases CO_2_ to the atmosphere.
Sediments: Material that has precipitated through the water column and settled on the bottom of a lake or ocean.
Primary production: Production of biomass by phototrophic organisms, such as phytoplankton or plants.
Bloom: The growth to high concentration certain types of microorganisms, such as phytoplankton; typically in the form of a boom and bust cycle which consists of the rapid cell division of phytoplankton followed by growth of, for example, a virus that lyses the cells and causes the collapse of the bloom.
Diatoms: A class (Bacillariophyceae) of single-celled algae that have a silica-containing skeleton.
Respiration: Heterotrophic respiration by microorganisms and autotrophic respiration by plants generate CO_2_, and photosynthetic respiration by plants, microalgae and cyanobacteria fixes CO_2_ and generates O_2_.
Methanogens: Anaerobic members of the Archaea that generate methane by methanogenesis. They reduce carbon dioxide, acetic acid or various one-carbon compounds, such as methylamines or methanol, to generate energy for growth.
Growth efficiency: A measure of how effectively microorganisms convert organic matter into biomass, with lower efficiency meaning more carbon is released to the atmosphere.
Oligotrophic: Conditions low in nutrients or nutrient flux, particularly of carbon, nitrogen or phosphorus, thereby limiting the concentration of cells the system supports; the bulk of the ocean is oligotrophic, apart from the coast and upwelling sites.
Cyanobacteria: Oxygen-producing photosynthetic bacteria that use sunlight as an energy source.
Microbial loop: The microbial component of a food web; for example, organic matter in marine microorganisms is released due to cell death and predation by grazers and viruses and used as nutrients for growth of cells that then feed higher trophic level organisms.
Photosynthesis: The conversion of sunlight into energy used to produce ATP and the subsequent fixing (conversion) of CO_2_ into organic matter; the process is photoautotrophic.
Autotrophic: Able to grow on carbon dioxide as the sole source of carbon.
Heterotrophic: Using organic compounds as nutrients to produce energy for growth.
Earth system model: A simulation of Earth’s physical (including climate), chemical and biological processes that integrates interactions of the biosphere with the atmosphere, ocean, land and ice.
Rhizosphere: The soil zone that surrounds and is influenced by the roots of plants.
Detritivores: Organisms that grow by decomposing detritus (animal and plant organic matter).
Denitrification: The process of converting oxidized forms of nitrogen such as nitrate (NO_3_) or nitrite (NO_2_) into more reduced forms, including nitrous oxide (N_2_O) and nitrogen gas (N_2_).
Forcings: Climate (or radiative) forcings are factors (for example, anthropogenic greenhouse gases, surface reflectivity (albedo), aerosols) other than the climate system itself (for example, oceans, land surface, cryosphere, biosphere and atmosphere) that cause climate change. Positive forcing occurs when more energy from sunlight is absorbed by Earth than is radiated back into space.

## References

[1] Barnosky AD (2011). Has the Earth’s sixth mass extinction already arrived?. Nature.

[2] Crist E, Mora C, Engelman R (2017). The interaction of human population, food production, and biodiversity protection. Science.

[3] Johnson CN (2017). Biodiversity losses and conservation responses in the Anthropocene. Science.

[4] Pecl GT (2017). Biodiversity redistribution under climate change: Impacts on ecosystems and human well-being. Science.

[5] Ripple WJ (2017). World scientists’ warning to humanity: a second notice. BioScience.

[6] United Nations Department of Economic and Social Affairs. *The Sustainable Development Goals Report** 2018* (United Nations, 2018).

[7] Timmis K (2019). The urgent need for microbiology literacy in society. Environ. Microbiol..

[8] Flemming HC, Wuertz S (2019). Bacteria and archaea on Earth and their abundance in biofilms. Nat. Rev. Microbiol..

[9] Maloy, S., Moran, M. A., Mulholland, M. R., Sosik, H. M. & Spear, J. R. *Microbes and Climate Change: Report on an American Academy of Microbiology and American Geophysical Union Colloquium held in Washington, DC, in March 2016* (American Society for Microbiology, 2017). 30063309

[10] Jørgensen BB, Boetius A (2007). Feast and famine — microbial life in the deep-sea bed. Nat. Microbiol. Rev..

[11] Sunagawa S (2015). Structure and function of the global ocean microbiome. Science.

[12] Karner MB, DeLong EF, Karl DM (2001). Archaeal dominance in the mesopelagic zone of the Pacific Ocean. Nature.

[13] Azam F, Malfatti F (2007). Microbial structuring of marine ecosystems. Nat. Rev. Microbiol..

[14] Kallmeyer J, Pockalny R, Adhikari RR, Smith DC, D’Hondt S (2012). Global distribution of microbial abundance and biomass in subseafloor sediment. Proc. Natl Acad. Sci. USA.

[15] Bar-On YM, Phillips R, Milo R (2018). The biomass distribution on Earth. Proc. Natl Acad. Sci. USA.

[16] Danovaro R, Corinaldesi C, Rastelli E, Dell’Anno A (2015). Towards a better quantitative assessment of the relevance of deep-sea viruses, Bacteria and Archaea in the functioning of the ocean seafloor. Aquat. Microb. Ecol..

[17] Caldeira K, Wickett ME (2003). Oceanography: anthropogenic carbon and ocean pH. Nature.

[18] Bunse C (2016). Response of marine bacterioplankton pH homeostasis gene expression to elevated CO_2_. Nat. Clim. Change.

[19] Hurd CL, Lenton A, Tilbrook B, Boyd PW (2018). Current understanding and challenges for oceans in a higher-CO2 world. Nat. Clim. Change.

[20] Hönisch B (2012). The geological record of ocean acidification. Science.

[21] Sosdian SM (2018). Constraining the evolution of Neogene ocean carbonate chemistry using the boron isotope pH proxy. Earth Planet. Sci. Lett..

[22] Riebesell U, Gattuso J-P (2015). Lessons learned from ocean acidification research. Nat. Clim. Change.

[23] Gao K (2012). Rising CO2 and increased light exposure synergistically reduce marine primary productivity. Nat. Clim. Change.

[24] Boyd PW (2013). Framing biological responses to a changing ocean. Nat. Clim. Change.

[25] Pörtner, H.-O. et al. in *Climate Change 2014 — Impacts, Adaptation and Vulnerability: Part A: Global and Sectoral Aspects: Working Group II Contribution to the IPCC Fifth Assessment Report* (eds Field, C. B. et al.) 411–484 (Cambridge University Press, 2014).

[26] Brennan G, Collins S (2015). Growth responses of a green alga to multiple environmental drivers. Nat. Clim. Change.

[27] Hutchins DA, Boyd PW (2016). Marine phytoplankton and the changing ocean iron cycle. Nat. Clim. Change.

[28] Hutchins DA, Fu FX (2017). Microorganisms and ocean global change. Nat. Microbiol..

[29] Rintoul SR (2018). Choosing the future of Antarctica. Nature.

[30] Behrenfeld MJ (2014). Climate-mediated dance of the plankton. Nat. Clim. Change.

[31] De Baar HJW (1995). Importance of iron for plankton blooms and carbon dioxide drawdown in the Southern Ocean. Nature.

[32] Boyd PW (2007). Mesoscale iron enrichment experiments 1993-2005: synthesis and future directions. Science.

[33] Behrenfeld MJ (2016). Revaluating ocean warming impacts on global phytoplankton. Nat. Clim. Change.

[34] Behrenfeld MJ (2017). Annual boom-bust cycles of polar phytoplankton biomass revealed by space-based lidar. Nat. Geosci..

[35] Behrenfeld MJ (2006). Climate-driven trends in contemporary ocean productivity. Nature.

[36] Levitan O (2007). Elevated CO2 enhances nitrogen fixation and growth in the marine cyanobacterium Trichodesmium. Glob. Change Biol..

[37] Verspagen JM, Van de Waal DB, Finke JF, Visser PM, Huisman J (2014). Contrasting effects of rising CO_2_ on primary production and ecological stoichiometry at different nutrient levels. Ecol. Lett..

[38] Holding JM (2015). Temperature dependence of CO2-enhanced primary production in the European Arctic Ocean. Nat. Clim. Change.

[39] Boyce DG, Lewis MR, Worm B (2010). Global phytoplankton decline over the past century. Nature.

[40] Mackas DL (2011). Does blending of chlorophyll data bias temporal trend?. Nature.

[41] Rykaczewski RR, Dunne JP (2011). A measured look at ocean chlorophyll trends. Nature.

[42] McQuatters-Gollop A (2011). Is there a decline in marine phytoplankton?. Nature.

[43] Boyce DG, Lewis MR, Worm B (2011). Boyce et al. reply. Nature.

[44] Antoine D, Morel A, Gordon HR, Banzon VF, Evans RH (2005). Bridging ocean color observations of the 1980s and 2000s in search of long-term trends. J. Geophys. Res. Oceans.

[45] Wernand MR, van der Woerd HJ, Gieskes WW (2013). Trends in ocean colour and chlorophyll concentration from 1889 to 2000, worldwide. PLOS ONE.

[46] Rousseaux CS, Gregg WW (2015). Recent decadal trends in global phytoplankton composition. Global Biogeochem. Cycles.

[47] Kirchman DL, Morán XA, Ducklow H (2009). Microbial growth in the polar oceans — role of temperature and potential impact of climate change. Nat. Rev. Microbiol..

[48] Dore JE, Lukas R, Sadler DW, Church MJ, Karl DM (2009). Physical and biogeochemical modulation of ocean acidification in the central North Pacific. Proc. Natl Acad. Sci. USA.

[49] Saba VS (2010). Challenges of modeling depth-integrated marine primary productivity over multiple decades: a case study at BATS and HOT. Global Biogeochem. Cycles.

[50] Buttigieg PL, Fadeev E, Bienhold C, Hehemann L, Offre P, Boetius A (2018). Marine microbes in 4D—using time series observation to assess the dynamics of the ocean microbiome and its links to ocean health. Curr. Opin. Microbiol..

[51] Rusch DB (2007). The Sorcerer II Global Ocean Sampling expedition: northwest Atlantic through eastern tropical Pacific. PLOS Biol..

[52] Brown MV (2012). Global biogeography of SAR11 marine bacteria. Mol. Syst. Biol..

[53] Wilkins D (2013). Biogeographic partitioning of Southern Ocean microorganisms revealed by metagenomics. Environ. Microbiol..

[54] Brum JR (2015). Patterns and ecological drivers of ocean viral communities. Science.

[55] de Vargas C (2015). Eukaryotic plankton diversity in the sunlit ocean. Science.

[56] Lima-Mendez G (2015). Determinants of community structure in the global plankton interactome. Science.

[57] Guidi L (2016). Plankton networks driving carbon export in the oligotrophic ocean. Nature.

[58] Roux S (2016). Ecogenomics and potential biogeochemical impacts of globally abundant ocean viruses. Nature.

[59] Gregory A (2019). Marine DNA viral macro- and micro-diversity from pole to pole. Cell.

[60] Nelson DM, Tréguer P, Brzezinski MA, Leynaert A, Quéguiner B (1995). Production and dissolution of biogenic silica in the ocean: revised global estimates, comparison with regional data and relationship to biogenic sedimentation. Global Biogeochem. Cycle.

[61] Malviya S (2016). Insights into global diatom distribution and diversity in the world’s ocean. Proc. Natl Acad. Sci. USA.

[62] Tréguer P (2018). Influence of diatom diversity on the ocean biological carbon pump. Nat. Geosci..

[63] Mahadevan A, D’Asaro E, Lee C, Perry MJ (2012). Eddy-driven stratification initiates North Atlantic spring phytoplankton blooms. Science.

[64] Boyd PW, Claustre H, Levy M, Siegel DA, Weber T (2019). Multi-faceted particle pumps drive carbon sequestration in the ocean. Nature.

[65] Behrenfeld MJ, Doney SC, Lima I, Boss ES, Siegel DA (2013). Annual cycles of ecological disturbance and recovery underlying the subarctic Atlantic spring plankton bloom. Global Biogeochem. Cycles.

[66] Field CB, Behrenfeld MJ, Randerson JT, Falkowski P (1998). Primary production of the biosphere: integrating terrestrial and oceanic components. Science.

[67] Behrenfeld MJ (2001). Biospheric primary production during an ENSO transition. Science.

[68] Boetius A (2013). Massive export of algal biomass from the melting Arctic sea ice. Science.

[69] Pachiadaki MG (2017). Major role of nitrite-oxidizing bacteria in dark ocean carbon fixation. Science.

[70] Grzymski JJ (2012). A metagenomic assessment of winter and summer bacterioplankton from Antarctic Peninsula coastal surface waters. ISME J..

[71] Boetius A, Wenzhöfer F (2013). Seafloor oxygen consumption fuelled by methane from cold seeps. Nat. Geosci..

[72] Danovaro R (2011). Marine viruses and global climate change. FEMS Microbiol. Rev..

[73] Schmidtko S, Stramma L, Visbeck M (2017). Decline in global oceanic oxygen content during the past five decades. Nature.

[74] Breitburg D (2018). Declining oxygen in the global ocean and coastal waters. Science.

[75] Bertagnolli AD, Stewart FJ (2018). Microbial niches in marine oxygen minimum zones. Nat. Rev. Microbiol..

[76] Danovaro R, Molari M, Corinaldesi C, Dell’Anno A (2016). Macroecological drivers of archaea and bacteria in benthic deep-sea ecosystems. Sci. Adv..

[77] Bienhold C, Zinger L, Boetius A, Ramette A (2016). Diversity and biogeography of bathyal and abyssal seafloor bacteria. PLOS ONE.

[78] Rosenfeld D (2019). Aerosol-driven droplet concentrations dominate coverage and water of oceanic low-level clouds. Science.

[79] Charlson RJ, Lovelock JE, Andreae MO, Warren SG (1987). Oceanic phytoplankton, atmospheric sulphur, cloud albedo and climate. Nature.

[80] Gantt B, Meskhidze N (2013). The physical and chemical characteristics of marine primary organic aerosol: a review. Atmos. Chem. Phys..

[81] Meskhidze N, Nenes A (2006). Phytoplankton and cloudiness in the Southern. Ocean. Science.

[82] Andreae MO, Rosenfeld D (2008). Aerosol–cloud–precipitation interactions. Part 1. The nature and sources of cloud-active aerosols. Earth Sci. Rev..

[83] Moore RH (2013). Droplet number uncertainties associated with CCN: an assessment using observations and a global model adjoint. Atmos. Chem. Phys..

[84] Sanchez KJ (2018). Substantial seasonal contribution of observed biogenic sulfate particles to cloud condensation nuclei. Sci. Rep..

[85] Atwood TB (2015). Predators help protect carbon stocks in blue carbon ecosystems. Nat. Clim. Change.

[86] Myers RA, Worm B (2003). Rapid worldwide depletion of predatory fish communities. Nature.

[87] Duarte CM, Losada IJ, Hendriks IE, Mazarrasa I, Marbà N (2013). The role of coastal plant communities for climate change mitigation and adaptation. Nat. Clim. Change.

[88] Hoffmann AA, Sgrò CM (2011). Climate change and evolutionary adaptation. Nature.

[89] Hughes TP (1994). Catastrophes, phase shifts, and large-scale degradation of a Caribbean coral reef. Science.

[90] Bellwood DR, Hoey AS, Ackerman JL, Depczynski M (2006). Coral bleaching, reef fish community phase shifts and the resilience of coral reefs. Glob. Change Biol..

[91] Hoegh-Guldberg O (2007). Coral reefs under rapid climate change and ocean acidification. Science.

[92] Mumby PJ, Hastings A, Edwards HJ (2007). Thresholds and the resilience of Caribbean coral reefs. Nature.

[93] Enochs IC (2015). Shift from coral to macroalgae dominance on a volcanically acidified reef. Nat. Clim. Change.

[94] De Bakker DM (2017). 40 years of benthic community change on the Caribbean reefs of Curaçao and Bonaire: the rise of slimy cyanobacterial mats. Coral Reefs.

[95] Ford AK (2018). Reefs under siege: the rise, putative drivers, and consequences of benthic cyanobacterial mats. Front. Mar. Sci..

[96] Ziegler M, Seneca FO, Yum LK, Palumbi SR, Voolstra CR (2017). Bacterial community dynamics are linked to patterns of coral heat tolerance. Nat. Commun..

[97] Torda G (2017). Rapid adaptive responses to climate change in corals. Nat. Clim. Change.

[98] Quigley, K. M., Baker, A. C., Coffroth, M. A., Willis, B. L. & van Oppen, M. J. H. in *Coral Bleaching: Patterns, Processes, Causes and Consequences* Ch. 6 (eds van Oppen, M. J. H. & Lough, J. M.) (Springer, 2018).

[99] Bourne DG, Morrow KM, Webster NS (2016). Insights into the coral microbiome: Underpinning the health and resilience of reef ecosystems. Annu. Rev. Microbiol..

[100] Webster NS, Reusch TBH (2017). Microbial contributions to the persistence of coral reefs. ISME J..

[101] Hanson CA, Fuhrman JA, Horner-Devine MC, Martiny JBH (2012). Beyond biogeographic patterns: processes shaping the microbial landscape. Nat. Rev. Microbiol..

[102] Zinger L, Boetius A, Ramette A (2014). Bacterial taxa-area and distance-decay relationships in marine environments. Mol. Ecol..

[103] Archer SDJ (2019). Airborne microbial transport limitation to isolated Antarctic soil habitats. Nat. Microbiol..

[104] Wilkins D, van Sebille E, Rintoul SR, Lauro FM, Cavicchioli R (2013). Advection shapes Southern Ocean microbial assemblages independent of distance and environment effects. Nat. Commun..

[105] Cavicchioli R (2015). Microbial ecology of Antarctic aquatic systems. Nat. Rev. Microbiol..

[106] Riebesell U (2018). Toxic algal bloom induced by ocean acidification disrupts the pelagic food web. Nat. Clim. Change.

[107] Hutchins DA (2015). Irreversibly increased nitrogen fixation in Trichodesmium experimentally adapted to elevated carbon dioxide. Nat. Commun..

[108] Schaum E, Rost B, Millar AJ, Sinéad C (2012). Variation in plastic responses to ocean acidification in a globally distributed picoplankton species. Nat. Clim. Change.

[109] Schlüter L (2014). Adaptation of a globally important coccolithophore to ocean warming and acidification. Nat. Clim. Change.

[110] Hoppe CJM, Wolf K, Schuback N, Tortell PD, Rost B (2018). Compensation of ocean acidification effects in Arctic phytoplankton assemblages. Nat. Clim. Change.

[111] Highfield A, Joint I, Gilbert JA, Crawfurd KJ, Schroeder DC (2017). Change in Emiliania huxleyi virus assemblage diversity but not in host genetic composition during an ocean acidification mesocosm experiment. Viruses.

[112] Flynn KJ (2012). Changes in pH at the exterior surface of plankton with ocean acidification. Nat. Clim. Change.

[113] Traving SJ, Clokie MR, Middelboe M (2014). Increased acidification has a profound effect on the interactions between the cyanobacterium Synechococcus sp. WH7803 and its viruses. FEMS Microbiol. Ecol..

[114] Follows MJ, Dutkiewicz S, Grant S, Chisholm SW (2007). Emergent biogeography of microbial communities in a model ocean. Science.

[115] Barton AD, Dutkiewicz S, Flierl G, Bragg J, Follows MJ (2010). Patterns of diversity in marine phytoplankton. Science.

[116] Thomas MK, Kremer CT, Klausmeier CA, Litchman EA (2012). Global pattern of thermal adaptation in marine phytoplankton. Science.

[117] Swan BK (2013). Prevalent genome streamlining and latitudinal divergence of surface ocean bacterioplankton. Proc. Natl Acad. Sci. USA.

[118] Barton AD, Irwin AJ, Finkel ZV, Stock CA (2016). Anthropogenic climate change drives shift and shuffle in North Atlantic phytoplankton communities. Proc. Natl Acad. Sci. USA.

[119] Cavicchioli R (2016). On the concept of a psychrophile. ISME J..

[120] Toseland A (2013). The impact of temperature on marine phytoplankton resource allocation and metabolism. Nat. Clim. Change.

[121] Moran XAG, Lopez-Urrutia A, Calvo-Diaz A, Li WKL (2010). Increasing importance of small phytoplankton in a warmer ocean. Glob. Change Biol..

[122] Thornton DCO (2014). Dissolved organic matter (DOM) release by phytoplankton in the contemporary and future ocean. Eur. J. Phycol..

[123] Jiang H-B (2018). Ocean warming alleviates iron limitation of marine nitrogen fixation. Nat. Clim. Change.

[124] Webster NS, Wagner M, Negri AP (2018). Microbial conservation in the Anthropocene. Environ. Microbiol..

[125] Cavicchioli R (2019). A vision for a ‘microbcentric’ future’. Microb. Biotechnol..

[126] Singh BK, Bardgett RD, Smith P, Reay DS (2010). Microorganisms and climate change: terrestrial feedbacks and mitigation options. Nat. Rev. Microbiol..

[127] Bardgett RD, van der Putten WH (2014). Belowground biodiversity and ecosystem functioning. Nature.

[128] Fellbaum CR, Mensah JA, Pfeffer PE, Kiers ET, Bücking H (2012). The role of carbon in fungal nutrient uptake and transport Implications for resource exchange in the arbuscular mycorrhizal symbiosis. Plant Signal. Behav..

[129] Ballantyne A (2017). Accelerating net terrestrial carbon uptake during the warming hiatus due to reduced respiration. Nat. Clim. Change.

[130] Bonan GB (2008). Forests and climate change: forcings, feedbacks, and the climate benefits of forests. Science.

[131] Pan Y (2011). A large and persistent carbon sink in the world’s forests. Science.

[132] Hovenden MJ (2019). Globally consistent influences of seasonal precipitation limit grassland biomass response to elevated CO2. Nat. Plants.

[133] Evans RD (2014). Greater ecosystem carbon in the Mojave Desert after ten years exposure to elevated CO_2_. Nat. Clim. Change.

[134] Verpoorter C, Kutser T, Seekell DA, Tranvik LJ (2014). A global inventory of lakes based on high-resolution satellite imagery. Geophys. Res. Lett..

[135] Davidson TA (2018). Synergy between nutrients and warming enhances methane ebullition from experimental lakes. Nat. Clim. Change.

[136] van Bergen TJHM (2019). Seasonal and diel variation in greenhouse gas emissions from an urban pond and its major drivers. Limnol. Oceanogr..

[137] Bragazza L, Parisod J, Buttler A, Bardgett RD (2013). Biogeochemical plant-soil microbe feedback in response to climate warming in peatlands. Nat. Clim. Change.

[138] Gallego-Sala AV, Prentice IC (2013). Blanket peat biome endangered by climate change. Nat. Clim. Change.

[139] Lupascu M (2014). High Arctic wetting reduces permafrost carbon feedbacks to climate warming. Nat. Clim. Change.

[140] Hultman J (2015). Multi-omics of permafrost, active layer and thermokarst bog soil microbiomes. Nature.

[141] Schuur EAG (2015). Climate change and the permafrost carbon feedback. Nature.

[142] Hoegh-Guldberg, O. et al. in *Special Report: Global Warming of 1.5°C* (eds Masson-Delmotte, V. et al.) Ch. 3 (IPCC, 2018).

[143] Crowther TW (2016). Quantifying global soil carbon losses in response to warming. Nature.

[144] Hicks Pries CE, Castanha C, Porras RC, Torn MS (2017). The whole-soil carbon flux in response to warming. Science.

[145] van Gestel N (2018). Predicting soil carbon loss with warming. Nature.

[146] Crowther TW (2018). Crowther et al. reply. Nature.

[147] Karhu K (2014). Temperature sensitivity of soil respiration rates enhanced by microbial community response. Nature.

[148] Norby RJ, Ledford J, Reilly CD, Miller NE, O’Neill EG (2004). Fine-root production dominates response of a deciduous forest to atmospheric CO_2_ enrichment. Proc. Natl Acad. Sci. USA.

[149] Lewis SL (2009). Increasing carbon storage in intact African tropical forests. Nature.

[150] Schlesinger WH, Lichter J (2001). Limited carbon storage in soil and litter of experimental forest plots under increased atmospheric CO_2_. Nature.

[151] Sayer EJ, Heard MS, Grant HK, Marthews TR, Tanner EVJ (2011). Soil carbon release enhanced by increased tropical forest litterfall. Nat. Clim. Change.

[152] Bradford MA (2016). Managing uncertainty in soil carbon feedbacks to climate change. Nat. Clim. Change.

[153] Hartley IP (2012). A potential loss of carbon associated with greater plant growth in the European Arctic. Nat. Clim. Change.

[154] Giardina CP, Litton CM, Crow SE, Asner GP (2014). Warming-related increases in soil CO2 efflux are explained by increased below-ground carbon flux. Nat. Clim. Change.

[155] Bradford MA (2014). Climate fails to predict wood decomposition at regional scales. Nat. Clim. Change.

[156] Fernández-Martínez M (2014). Nutrient availability as the key regulator of global forest carbon balance. Nat. Clim. Change.

[157] Högberg P (2001). Large-scale forest girdling shows that current photosynthesis drives soil respiration. Nature.

[158] Clemmensen KE (2013). Roots and associated fungi drive long-term carbon sequestration in boreal forest. Science.

[159] Keiluweit M (2015). Mineral protection of soil carbon counteracted by root exudates. Nat. Clim. Change.

[160] Tang J, Riley WJ (2015). Weaker soil carbon–climate feedbacks resulting from microbial and abiotic interactions. Nat. Clim. Change.

[161] Schmidt MW (2011). Persistence of soil organic matter as an ecosystem property. Nature.

[162] Sulman BN, Phillips RP, Oishi AC, Shevliakova E, Pacala SW (2014). Microbe-driven turnover offsets mineral-mediated storage of soil carbon under elevated CO_2_. Nat. Clim. Change.

[163] Stevnbak K (2012). Interactions between above- and belowground organisms modified in climate change experiments. Nat. Clim. Change.

[164] Bardgett RD, Wardle DA (2003). Herbivore-mediated linkages between aboveground and belowground communities. Ecology.

[165] Lubbers IM (2013). Greenhouse-gas emissions from soils increased by earthworms. Nat. Clim. Change.

[166] Thakur MP (2018). Reduced feeding activity of soil detritivores under warmer and drier conditions. Nat. Clim. Change.

[167] Hodgkins SB (2018). Tropical peatland carbon storage linked to global latitudinal trends in peat recalcitrance. Nat. Commun..

[168] Jansson JK, Tas N (2014). The microbial ecology of permafrost. Nat. Rev. Microbiol..

[169] McCalley CK (2014). Methane dynamics regulated by microbial community response to permafrost thaw. Nature.

[170] Grosse G, Goetz S, McGuire AD, Romanovsky VE, Schuur EAG (2016). Changing permafrost in a warming world and feedbacks to the Earth system. Environ. Res. Lett..

[171] Hicks Pries CE, Schuur EAG, Natali SM, Crummer KG (2016). Old soil carbon losses increase with ecosystem respiration in experimentally thawed tundra. Nat. Clim. Change.

[172] Knoblauch C, Beer C, Liebner S, Grigoriev MN, Pfeiffer E-M (2018). Methane production as key to the greenhouse gas budget of thawing permafrost. Nat. Clim. Change.

[173] Jing X (2015). The links between ecosystem multifunctionality and above- and belowground biodiversity are mediated by climate. Nat. Commun..

[174] Delgado-Baquerizo M (2016). Microbial diversity drives multifunctionality in terrestrial ecosystems. Nat. Commun..

[175] Walker TWN (2018). Microbial temperature sensitivity and biomass change explain soil carbon loss with warming. Nat. Clim. Change.

[176] Zhou JZ (2012). Microbial mediation of carbon-cycle feedbacks to climate warming. Nat. Clim. Change.

[177] Zhou J (2016). Temperature mediates continental-scale diversity of microbes in forest soils. Nat. Commun..

[178] Guo X (2018). Climate warming leads to divergent succession of grassland microbial communities. Nat. Clim. Change.

[179] Bradford MA (2019). Cross-biome patterns in soil microbial respiration predictable from evolutionary theory on thermal adaptation. Nat. Ecol. Evol..

[180] Dacal M, Bradford MA, Plaza C, Maestre FT, García-Palacios P (2019). Soil microbial respiration adapts to ambient temperature in global drylands. Nat. Ecol. Evol..

[181] Lipson DA (2015). The complex relationship between microbial growth rate and yield and its implications for ecosystem processes. Front. Microbiol..

[182] Frey SD, Lee J, Melillo JM, Six J (2013). The temperature response of soil microbial efficiency and its feedback to climate. Nat. Clim. Change.

[183] Hagerty SB (2014). Accelerated microbial turnover but constant growth efficiency with warming in soil. Nat. Clim. Change.

[184] Melillo J (2017). Long-term pattern and magnitude of soil carbon feedback to the climate system in a warming world. Science.

[185] Wieder WR, Bonan GB, Allison SD (2013). Global soil carbon projections are improved by modelling microbial processes. Nat. Clim. Change.

[186] Koven CD, Hugelius G, Lawrence DM, Wieder WR (2017). Higher climatological temperature sensitivity of soil carbon in cold than warm climates. Nat. Clim. Change.

[187] Mackelprang R, Saleska SR, Jacobsen CS, Jansson JK, Tas N (2016). Permafrost meta-omics and climate change. Annu. Rev. Earth Planet. Sci..

[188] Tas N (2018). Landscape topography structures the soil microbiome in arctic polygonal tundra. Nat. Commun..

[189] Woodcroft BJ (2018). Genome-centric view of carbon processing in thawing permafrost. Nature.

[190] Emerson JB (2018). Host-linked soil viral ecology along a permafrost thaw gradient. Nat. Microbiol..

[191] Singleton CM (2018). Methanotrophy across a natural permafrost thaw environment. ISME J..

[192] Xue K (2016). Tundra soil carbon is vulnerable to rapid microbial decomposition under climate warming. Nat. Clim. Change.

[193] Kane ES (2012). Squeezing the arctic carbon balloon. Nat. Clim. Change.

[194] Hill PW (2011). Vascular plant success in a warming Antarctic may be due to efficient nitrogen acquisition. Nat. Clim. Change.

[195] Newsham KK (2016). Relationship between soil fungal diversity and temperature in the maritime Antarctic. Nat. Clim. Change.

[196] Kleinteich J (2012). Temperature-related changes in polar cyanobacterial mat diversity and toxin production. Nat. Clim. Change.

[197] Paerl HW, Huisman J (2008). Blooms like it hot. Science.

[198] Huisman J (2018). Cyanobacterial blooms. Nat. Rev. Microbiol..

[199] Sitoki L, Kurmayer R, Rott E (2012). Spatial variation of phytoplankton composition, biovolume, and resulting microcystin concentrations in the Nyanza Gulf (Lake Victoria, Kenya). Hydrobiologia.

[200] Metcalf JS (2018). Public health responses to toxic cyanobacterial blooms: perspectives from the 2016 Florida event. Water Policy.

[201] Visser PM (2016). How rising CO_2_ and global warming may stimulate harmful cyanobacterial blooms. Harmful Algae.

[202] Walsby AE, Hayes PK, Boje R, Stal LJ (1997). The selective advantage of buoyancy provided by gas vesicles for planktonic cyanobacteria in the Baltic Sea. New Phytol..

[203] Jöhnk KD (2008). Summer heatwaves promote blooms of harmful cyanobacteria. Glob. Chang. Biol..

[204] Lehman PW (2017). Impacts of the 2014 severe drought on the Microcystis bloom in San Francisco Estuary. Harmful Algae.

[205] Sandrini G (2016). Rapid adaptation of harmful cyanobacteria to rising CO_2_. Proc. Natl Acad. Sci. USA.

[206] Lanz B, Dietz S, Swanson T (2018). The expansion of modern agriculture and global biodiversity decline: an integrated assessment. Ecol. Econ..

[207] Dai Z (2018). Long-term nitrogen fertilization decreases bacterial diversity and favors the growth of Actinobacteria and Proteobacteria in agro-ecosystems across the globe. Glob. Change Biol..

[208] Gålfalk M, Olofsson G, Crill P, Bastviken D (2016). Making methane visible. Nat. Clim. Change.

[209] Nisbet EG (2019). Very strong atmospheric methane growth in the four years 2014–2017: implications for the Paris Agreement. Global Biogeochem. Cycles.

[210] van Groenigen KS, van Kessel C, Hungate B, A. (2013). Increased greenhouse-gas intensity of rice production under future atmospheric conditions. Nat. Clim. Change.

[211] Ripple WJ (2014). Ruminants, climate change and climate policy. Nat. Clim. Change.

[212] Steffen W (2015). Sustainability. Planetary boundaries: guiding human development on a changing planet. Science.

[213] Greaver TL (2016). Key ecological responses to nitrogen are altered by climate change. Nat. Clim. Change.

[214] Itakura M (2013). Mitigation of nitrous oxide emissions from soils by Bradyrhizobium japonicum inoculation. Nat. Clim. Change.

[215] Godfray HC (2010). Food security: the challenge of feeding 9 billion people. Science.

[216] de Vries FT (2012). Land use alters the resistance and resilience of soil food webs to drought. Nat. Clim. Change.

[217] de Vries FT (2018). Soil bacterial networks are less stable under drought than fungal networks. Nat. Commun..

[218] Bahram M (2018). Structure and function of the global topsoil microbiome. Nature.

[219] Maestre FT (2015). Increasing aridity reduces soil microbial diversity and abundance in global drylands. Proc. Natl Acad. Sci. USA.

[220] Posch T, Köster O, Salcher MM, Pernthaler J (2012). Harmful filamentous cyanobacteria favoured by reduced water turnover with lake warming. Nat. Clim. Change.

[221] Harvell CD (2002). Climate warming and disease risks for terrestrial and marine biota. Science.

[222] Altizer S, Ostfeld RS, Johnson PT, Kutz S, Harvell CD (2013). Climate change and infectious diseases: from evidence to a predictive framework. Science.

[223] Johnson PTJ, de Roode JC, Fenton A (2015). Why infectious disease research needs community ecology. Science.

[224] Bruno JF (2007). Thermal stress and coral cover as drivers of coral disease outbreaks. PLOS Biol..

[225] Randall J, van Woesik R (2015). Contemporary white-band disease in Caribbean corals driven by climate change. Nat. Clim. Change.

[226] Maynard J (2015). Projections of climate conditions that increase coral disease susceptibility and pathogen abundance and virulence. Nat. Clim. Change.

[227] Randall CJ, van Woesik R (2017). Some coral diseases track climate oscillations in the Caribbean. Sci. Rep..

[228] Frommel AY (2012). Severe tissue damage in Atlantic cod larvae under increasing ocean acidification. Nat. Clim. Change.

[229] Harvell CD (2019). Disease epidemic and a marine heat wave are associated with the continental-scale collapse of a pivotal predator (Pycnopodia helianthoides). Sci. Adv..

[230] Ling SD (2015). Global regime shift dynamics of catastrophic sea urchin overgrazing. Phil. Trans. R. Soc. B.

[231] Maynard J (2016). Improving marine disease surveillance through sea temperature monitoring, outlooks and projections. Phil. Trans. R. Soc. B Biol. Sci..

[232] Anderegg WRL, Kane JM, Anderegg LDL (2013). Consequences of widespread tree mortality triggered by drought and temperature stress. Nat. Clim. Change.

[233] Bebber DP, Ramotowski MAT, Gurr SJ (2013). Crop pests and pathogens move polewards in a warming world. Nat. Clim. Change.

[234] Raffel TR (2013). Disease and thermal acclimation in a more variable and unpredictable climate. Nat. Clim. Change.

[235] Pounds JA (2006). Widespread amphibian extinctions from epidemic disease driven by global warming. Nature.

[236] MacFadden DR, McGough SF, Fisman D, Santillana M, Brownstein JS (2018). Antibiotic resistance increases with local temperature. Nat. Clim. Change.

[237] Patz JA, Campbell-Lendrum D, Holloway T, Foley JA (2005). Impact of regional climate change on human health. Nature.

[238] Semenza JC, Domanovic D (2013). Blood supply under threat. Nat. Clim. Change.

[239] Semenza JC (2012). Climate change impact assessment of food- and waterborne diseases. Crit. Rev. Environ. Sci. Technol..

[240] McIntyre KM (2017). Systematic assessment of the climate sensitivity of important human and domestic animals pathogens in Europe. Sci. Rep..

[241] Jones AE (2019). Bluetongue risk under future climates. Nat. Clim. Change.

[242] Baker-Austin C (2013). Emerging Vibrio risk at high latitudes in response to ocean warming. Nat. Clim. Change.

[243] Pascual M, Rodó X, Ellner SP, Colwell R, Bouma MJ (2000). Cholera dynamics and El Niño-Southern Oscillation. Science.

[244] Vezzulli L (2016). Climate influence on Vibrio and associated human diseases during the past half-century in the coastal North Atlantic. Proc. Natl Acad. Sci. USA.

[245] Bhatt S (2013). The global distribution and burden of dengue. Nature.

[246] Powell JR (2016). Mosquitoes on the move. Science.

[247] Lessler J (2016). Assessing the global threat from Zika virus. Science.

[248] Scheffers BR (2016). The broad footprint of climate change from genes to biomes to people. Science.

[249] Weaver SC (2018). Prediction and prevention of urban arbovirus epidemics: a challenge for the global virology community. Antiviral Res..

[250] Bouma MJ, Dye C (1997). Cycles of malaria associated with El Niño in Venezuela. JAMA.

[251] Baylis M, Mellor PS, Meiswinkel R (1999). Horse sickness and ENSO in South Africa. Nature.

[252] Rohani P (2009). The link between dengue incidence and El Nino Southern Oscillation. PLOS Med..

[253] Kreppel KS (2014). A non-stationary relationship between global climate phenomena and human plague incidence in Madagascar. PLOS Neglect. Trop. Dis..

[254] Caminade C (2017). Global risk model for vector-borne transmission of Zika virus reveals the role of El Niño 2015. Proc. Natl Acad. Sci. USA.

[255] Giraud T, Koskella B, Laine A-L (2017). Introduction: microbial local adaptation: insights from natural populations, genomics and experimental evolution. Mol. Ecol..

[256] Croll D, McDonald BA (2017). The genetic basis of local adaptation for pathogenic fungi in agricultural ecosystems. Molec. Ecol..

[257] Robin C, Andanson A, Saint-Jean G, Fabreguettes O, Dutech C (2017). What was old is new again: thermal adaptation within clonal lineages during range expansion in a fungal pathogen. Mol. Ecol..

[258] King JG, Souto-Maior C, Sartori LM, Maciel-de-Freitas R, Gomes MGM (2018). Variation in Wolbachia effects on Aedes mosquitoes as a determinant of invasiveness and vectorial capacity. Nat. Commun..

[259] Bakken LR, Frostegård Å (2017). Sources and sinks for N_2_O, can microbiologist help to mitigate N_2_O emissions?. Environ. Microbiol..

[260] Henderson G (2015). Rumen microbial community composition varies with diet and host, but a core microbiome is found across a wide geographical range. Sci. Rep..

[261] Roehe R (2016). Bovine host genetic variation influences rumen microbial methane production with best selection criterion for low methane emitting and efficiently feed converting hosts based on metagenomic gene abundance. PLOS Genet..

[262] Ritchie H, Reay DS, Higgins P (2018). Potential of meat substitutes for climate change mitigation and improved human health in high-income markets. Front. Sustain. Food Syst..

[263] Weng ZH (2017). Biochar built soil carbon over a decade by stabilizing rhizodeposits. Nat. Clim. Change.

[264] Liu D (2012). Constructed wetlands as biofuel production systems. Nat. Clim. Change.

[265] Sánchez O (2017). Constructed wetlands revisited: microbial diversity in the –omics era. Microb. Ecol..

[266] Timmis K (2017). The contribution of microbial biotechnology to sustainable development goals. Microb. Biotechnol..

[267] Union of Concerned Scientists. World scientists’ warning to humanity. *UCSUSA* http://www.ucsusa.org/sites/default/files/attach/2017/11/World%20Scientists%27%20Warning%20to%20Humanity%201992.pdf (1992).

[268] Ripple WJ (2018). The role of Scientists’ Warning in shifting policy from growth to conservation economy. BioScience.

[269] Finlayson CM (2019). The Second Warning to Humanity — providing a context for wetland management and policy. Wetlands.

[270] Colwell, R. R. & Patz, J. A. *Climate, Infectious Disease and Health: An Interdisciplinary Perspective* (American Academy of Microbiology, 1998). 32687286

[271] Reid, A. *Incorporating Microbial Processes Into Climate Models* (American Academy of Microbiology, 2012). 32865933

[272] Reid, A. & Greene, S. *How Microbes Can Help Feed The World* (American Academy of Microbiology, 2013).

[273] Paull SH (2017). Drought and immunity determine the intensity of West Nile virus epidemics and climate change impacts. Proc. R. Soc. B.

[274] Paaijmans KP (2010). Influence of climate on malaria transmission depends on daily temperature variation. Proc. Natl Acad. Sci. USA.

[275] Colón-González FJ (2018). Limiting global-mean temperature increase to 1.5–2°C could reduce the incidence and spatial spread of dengue fever in Latin America. Proc. Natl Acad. Sci. USA.

[276] Ostfeld RS, Brunner JL (2015). Climate change and *Ixodes* tick-borne diseases of humans. Philos. Trans. R. Soc. B.

[277] Moore SM (2017). El Niño and the shifting geography of cholera in Africa. Proc. Natl Acad. Sci. USA.

[278] Peng X, Murphy T, Holden NM (2008). Evaluation of the effect of temperature on the die-off rate for Cryptosporidium parvum oocysts in water, soils, and feces. Appl. Environ. Microbiol..

[279] Atchison CJ (2010). Temperature-dependent transmission of rotavirus in Great Britain and The Netherlands. Proc. R. Soc. Biol. B.

[280] Shaman J, Lipsitch M (2013). The El Niño–Southern Oscillation (ENSO)–pandemic Influenza connection: coincident or causal?. Proc. Natl Acad. Sci. USA.

[281] Shaman J, Karspeck A (2012). Forecasting seasonal outbreaks of influenza. Proc. Natl Acad. Sci. USA.

[282] Nguyen C (2013). Recent advances in our understanding of the environmental, epidemiological, immunological, and clinical dimensions of coccidioidomycosis. Clin. Microbiol. Rev..

[283] Tian H (2017). Interannual cycles of Hantaan virus outbreaks at the human–animal interface in Central China are controlled by temperature and rainfall. Proc. Natl Acad. Sci. USA.

[284] Glass GE (2002). Satellite imagery characterizes local animal reservoir populations of Sin Nombre virus in the southwestern United States. Proc. Natl Acad. Sci. USA.

